# Supplementary material for: Design, Synthesis, and Anticancer Activity Studies of Novel Quinoline-Chalcone Derivatives
Source: Molecules. 2021 Aug 13;26(16):4899. doi: 10.3390/molecules26164899 (PMC8398129; doi:10.3390/molecules26164899)
Supplement: Supplementary file 1 [file molecules-26-04899-s001.zip › molecules-1299243-SI.pdf]

Design, Synthesis and Anticancer Activity Studies of Novel Quinoline-Chalcone Derivatives

Yong-Feng Guan [a], Xiu-Juan Liu [c], Xin-Ying Yuan [c], Wen-Bo Liu [c], Yin-Ru Li [b], Guang-Xi Yu [b], Xin-Yi Tian [b], Yan-Bing Zhang [c], Jian Song [c, *], Wen Li [a, c, *], Sai-Yang Zhang [b, *]

a. School of Chemical Engineering, Zhengzhou University, Zhengzhou 450001, China.

b. School of Basic Medical Sciences, Zhengzhou University, Zhengzhou, 450001, China

c. School of Pharmaceutical Sciences, Institute of Drug Discovery & Development, Key Laboratory of Advanced Drug Preparation Technologies (Ministry of Education), Zhengzhou University, Zhengzhou, 450001, China

* Corresponding authors: mumuangzz@163.com (Jian Song), liwen@zzu.edu.cn (Wen Li), saiyangz@zzu.edu.cn (Sai-Yang Zhang)

^{1}H NMR of compound 12a

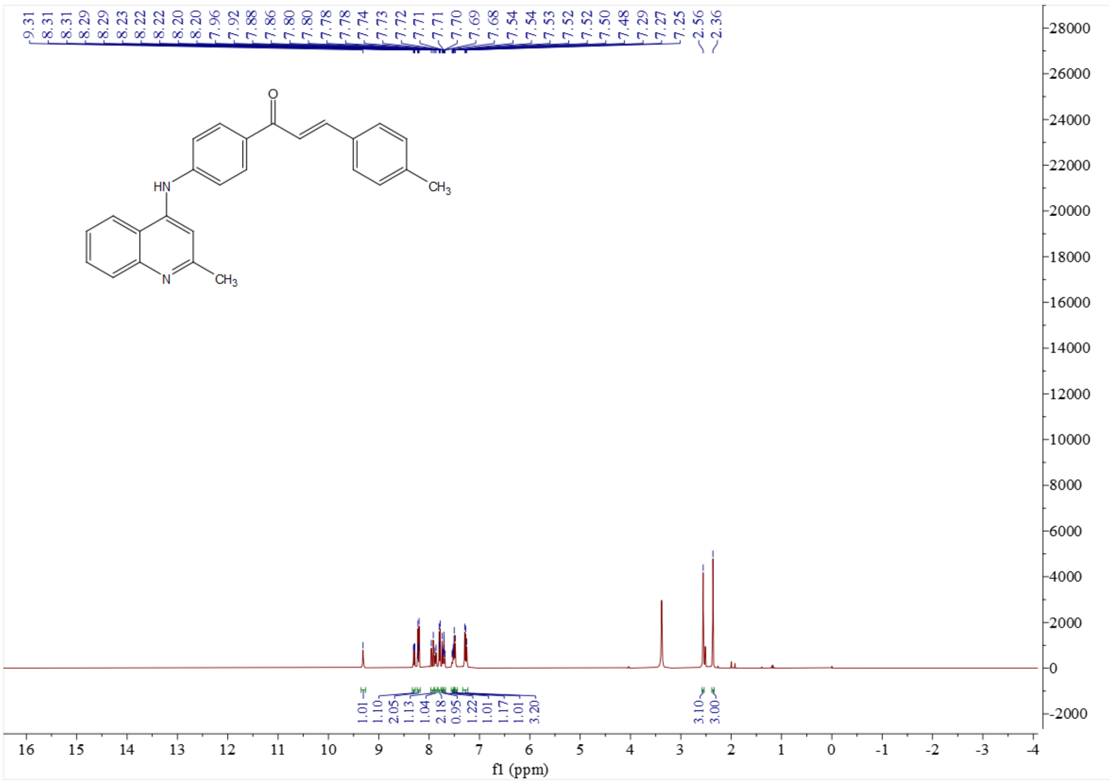

Figure S1. ^{1}H NMR spectrum of compound **12a**

^{13}C NMR of compound 12a

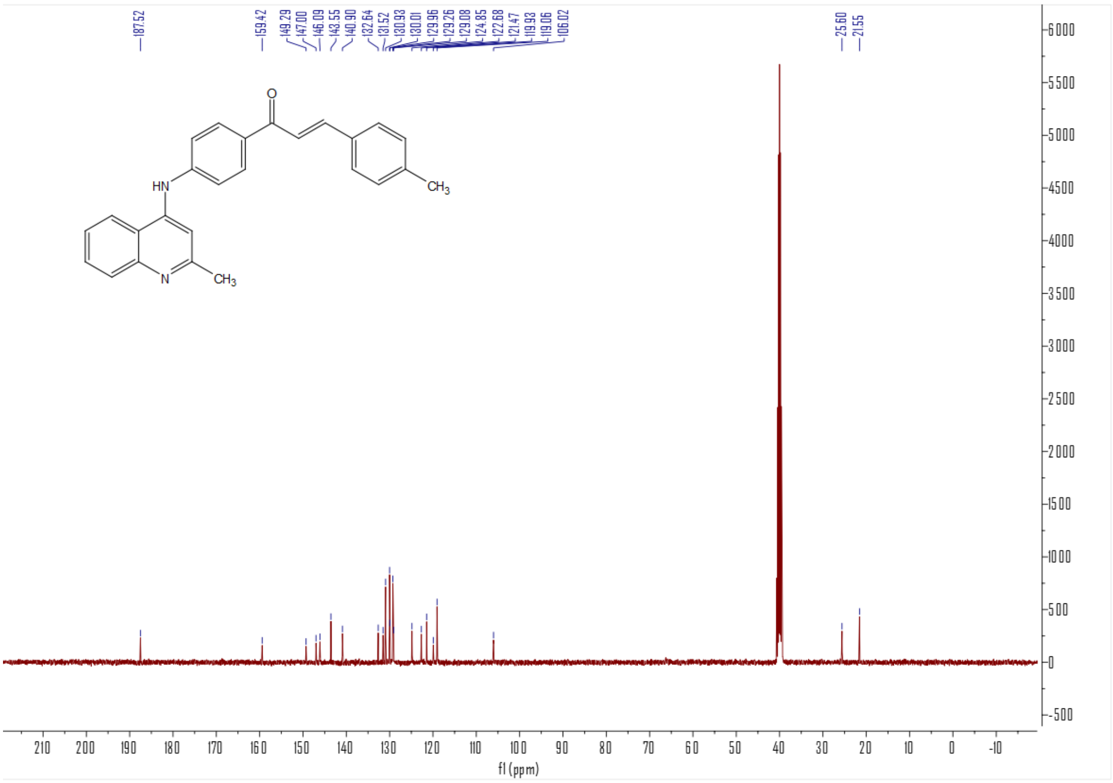

Figure S2 ^{13}C NMR spectrum of compound **12a**

HRMS spectrum of compound 12a

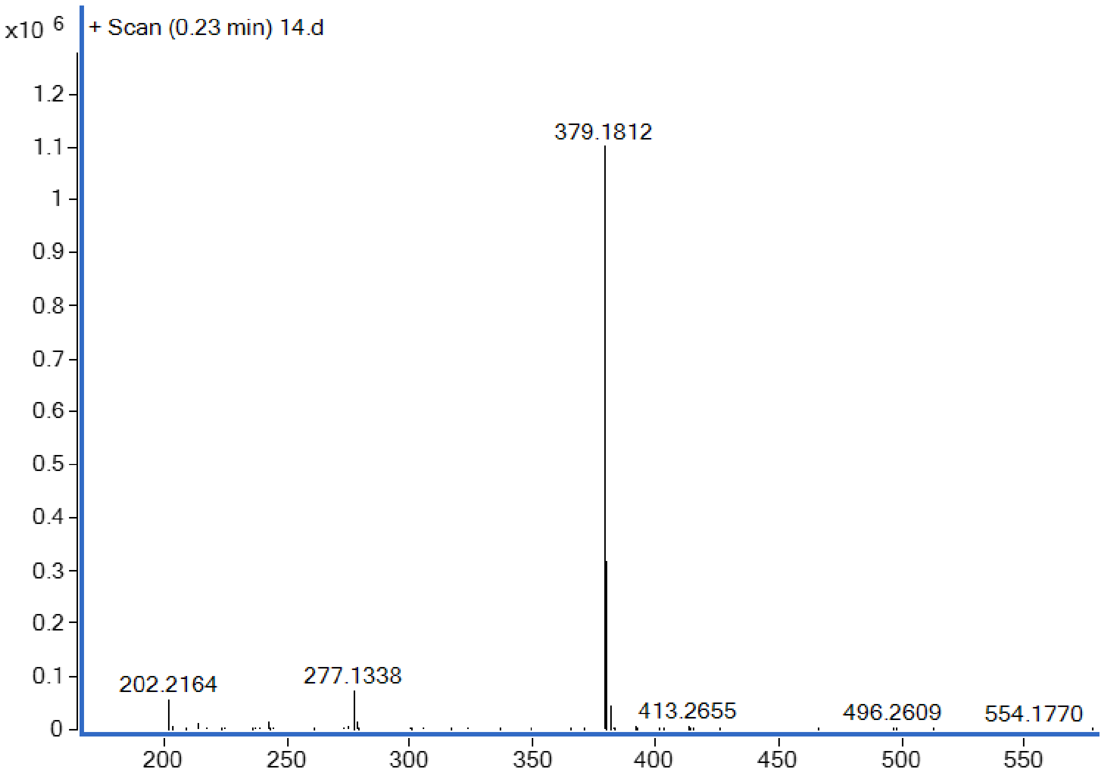

Figure S3 HRMS spectrum of compound **12a**

^{1}H NMR of compound 12b

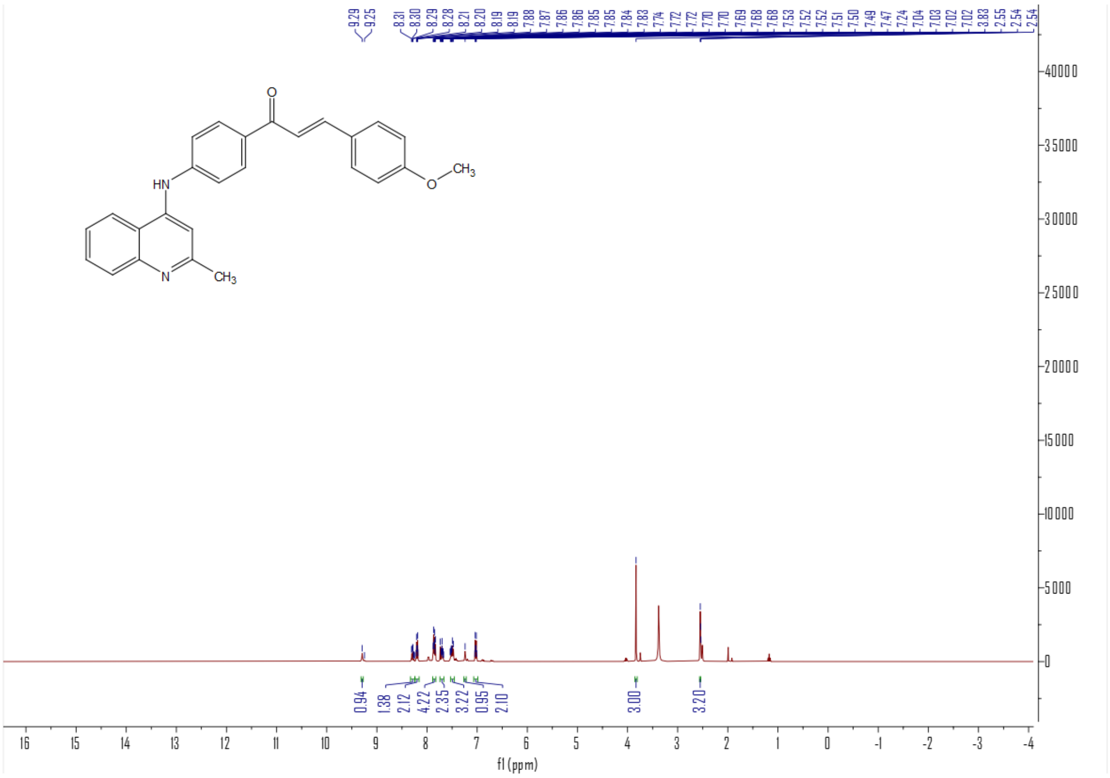

Figure S4 ^{1}H NMR spectrum of compound **12b**

^{13}C NMR of compound 12b

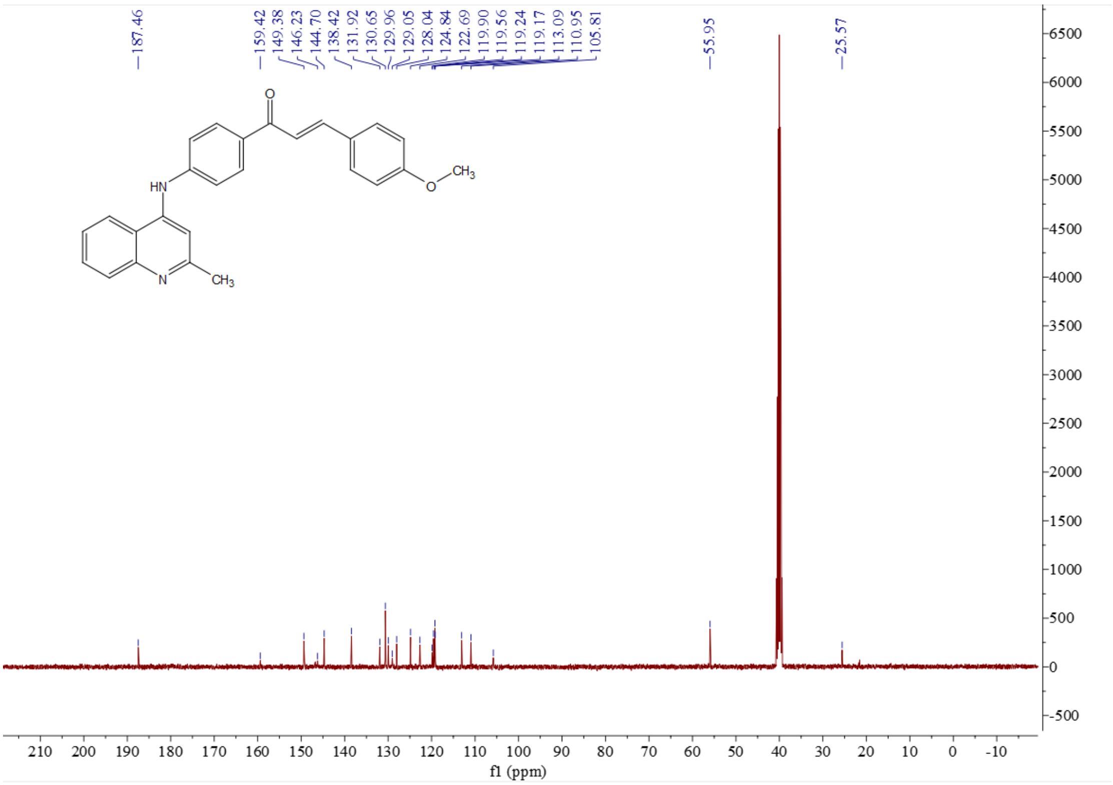

Figure S5 ^{13}C NMR spectrum of compound **12b**

HRMS spectrum of compound 12b

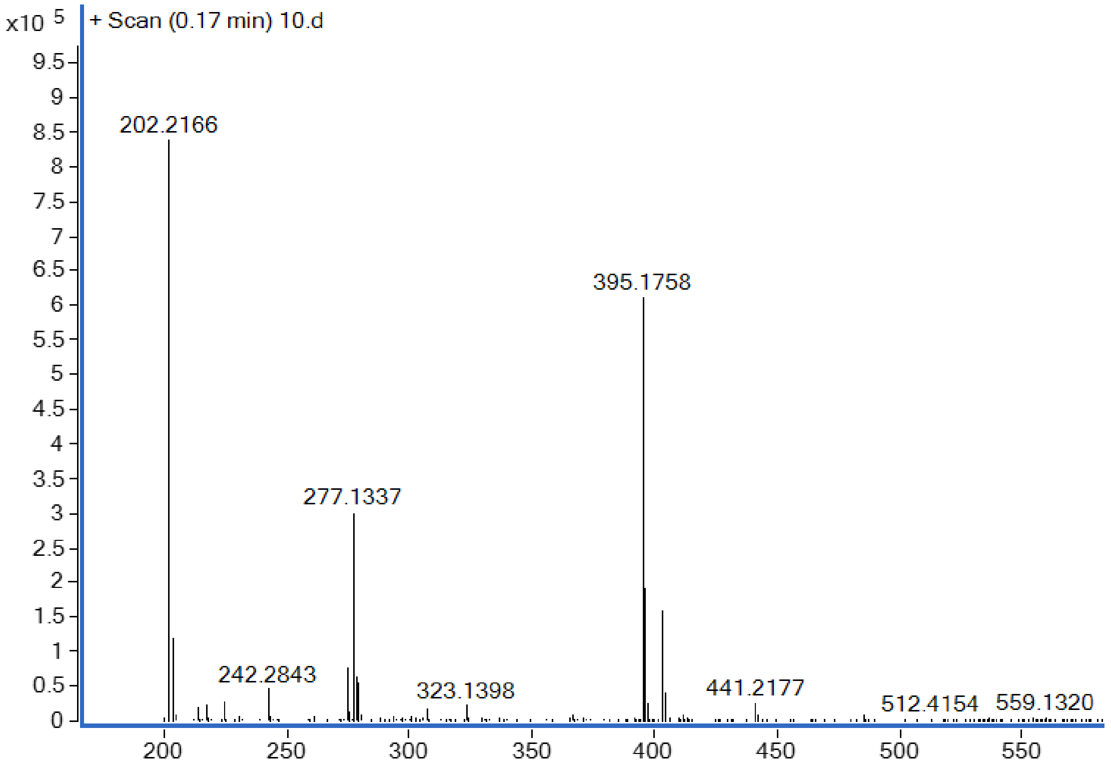

Figure S6 HRMS spectrum of compound **12b**

^{1}H NMR of compound 12c

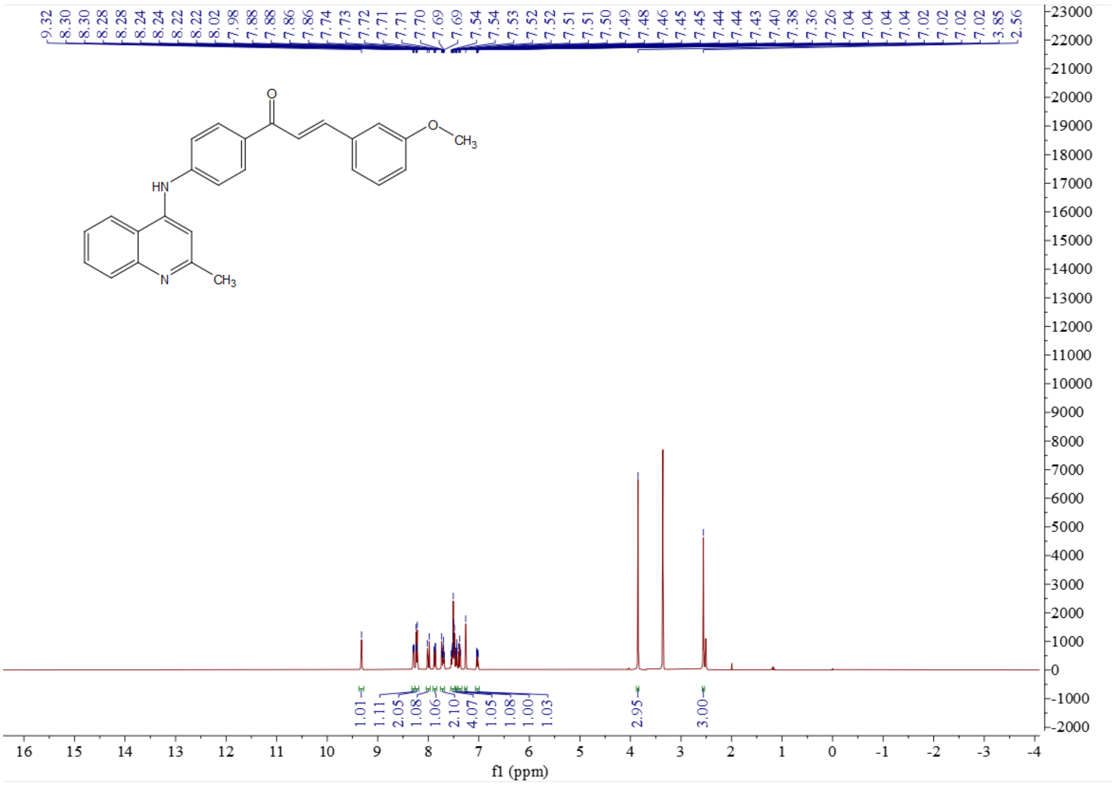

Figure S7 ^{1}H NMR spectrum of compound **12c**

^{13}C NMR of compound 12c

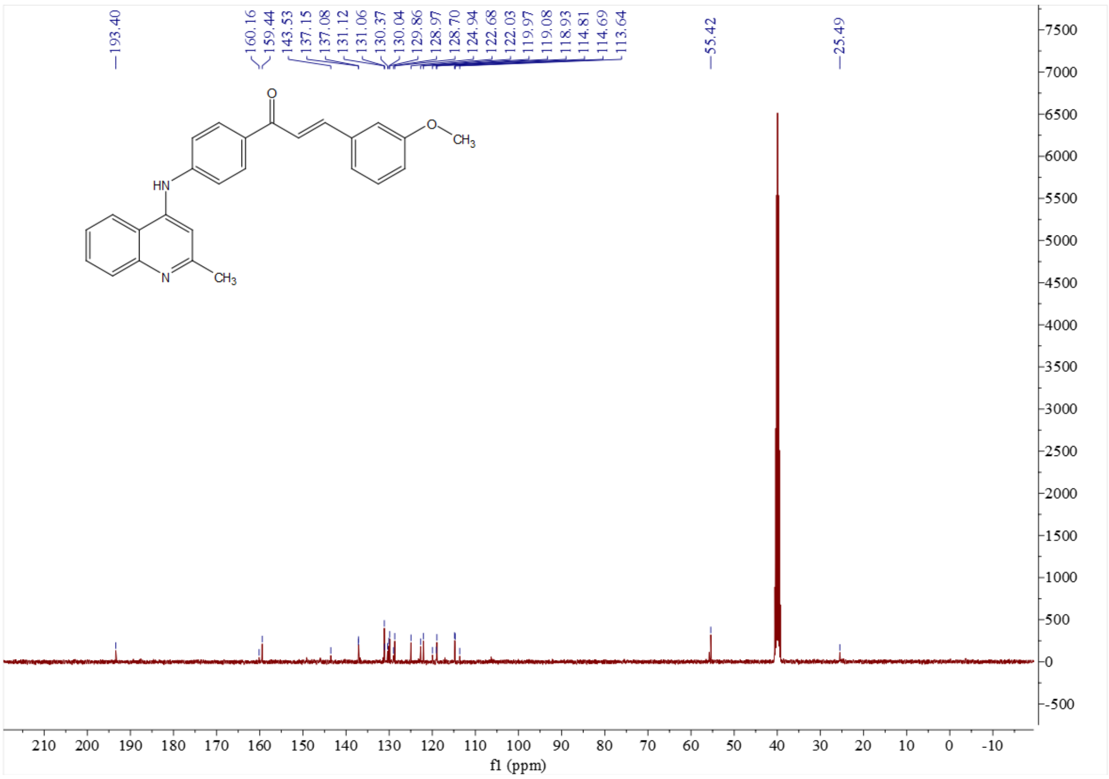

Figure S8 ^{13}C NMR spectrum of compound **12c**

HRMS spectrum of compound 12c

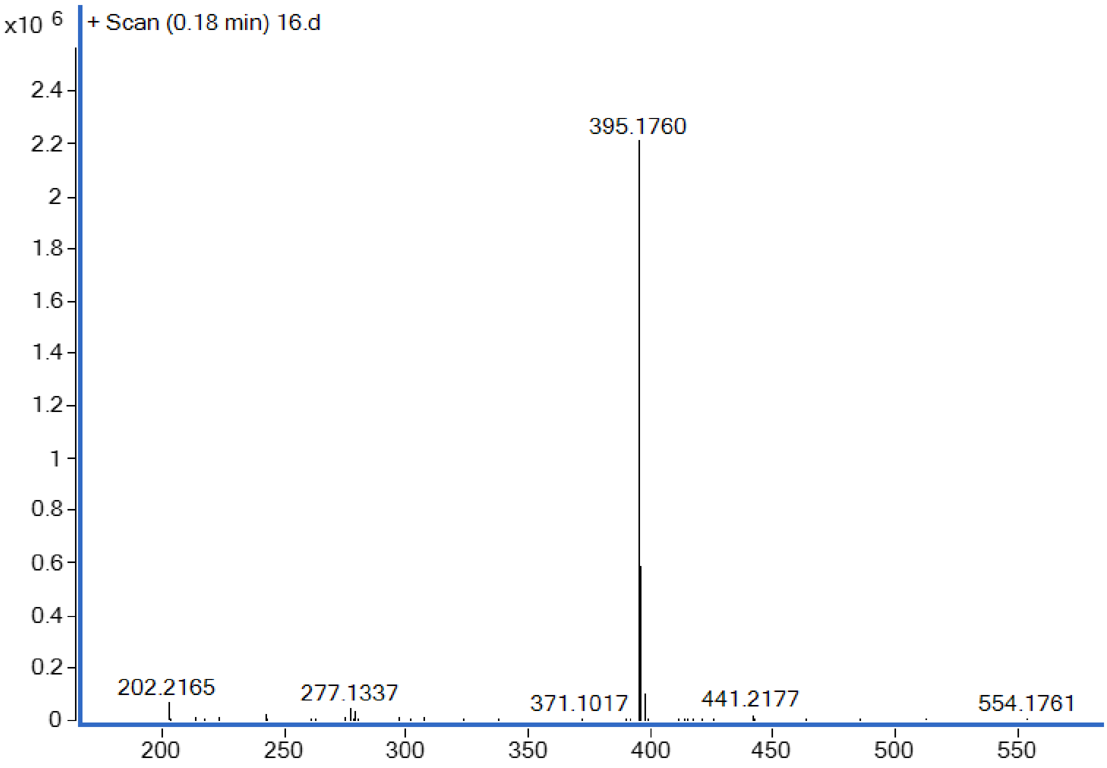

Figure S9 HRMS spectrum of compound **12c**

^{1}H NMR of compound 12d

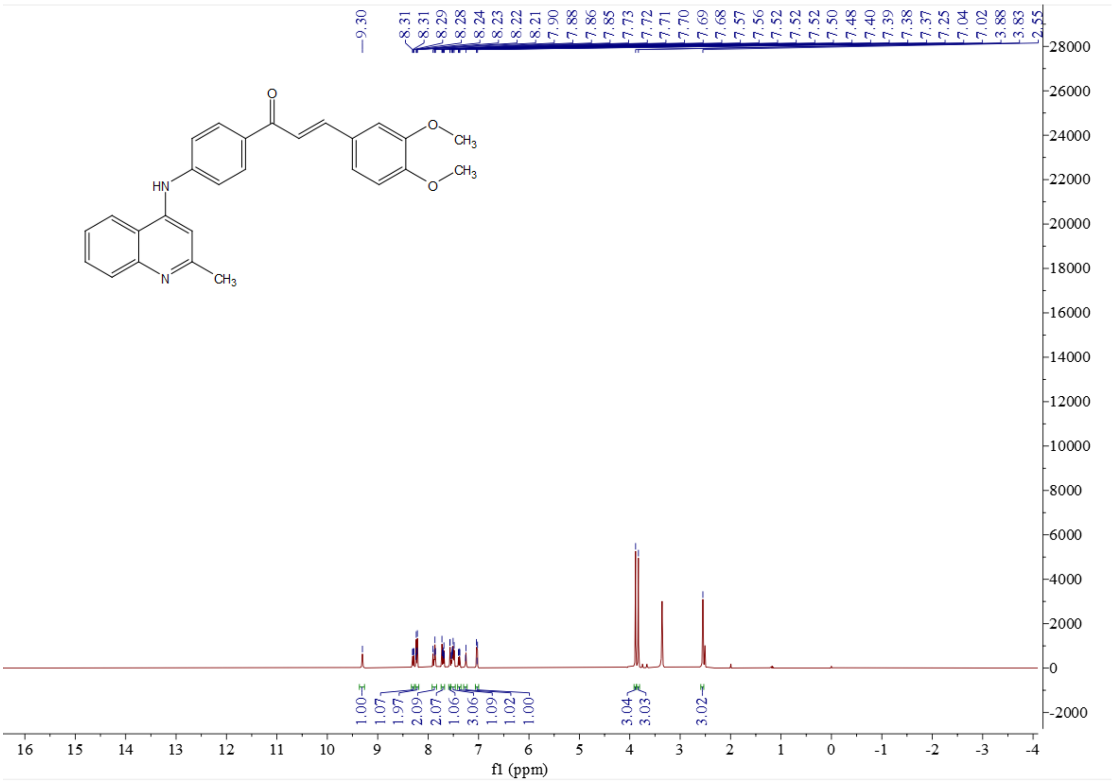

Figure S10 ^{1}H NMR spectrum of compound **12d**

^{13}C NMR of compound 12d

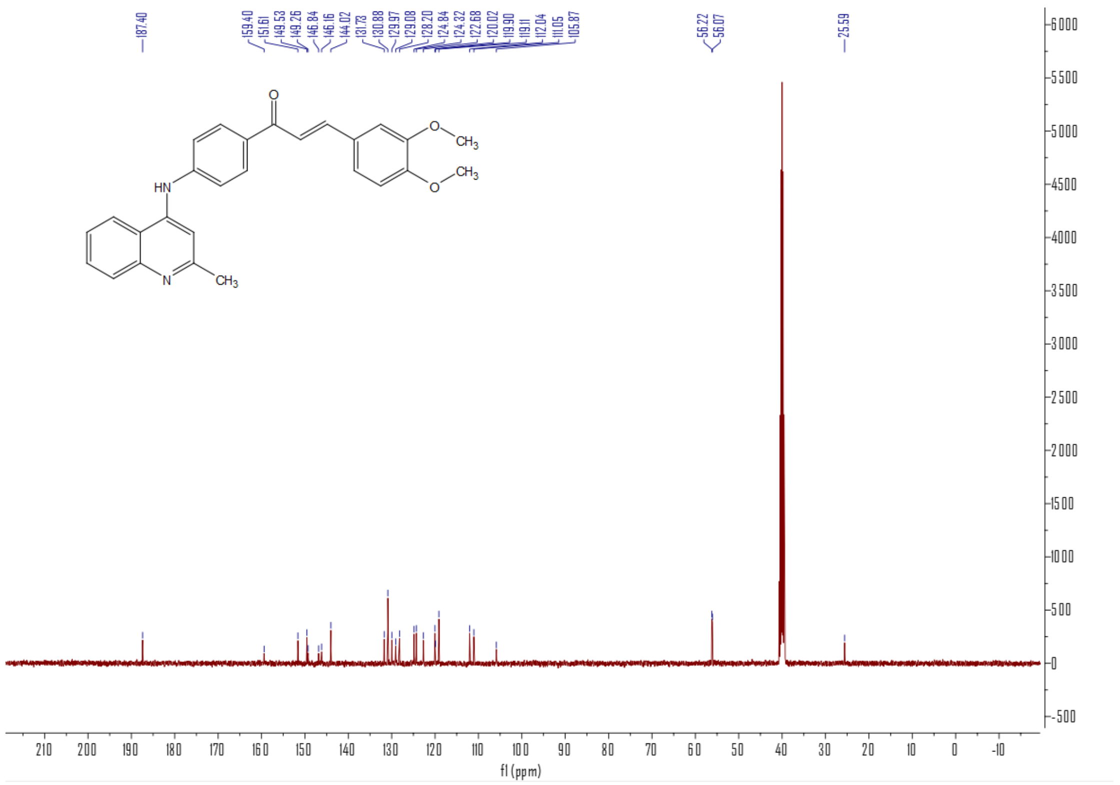

Figure S11 ^{13}C NMR spectrum of compound **12d**

HRMS spectrum of compound 12d

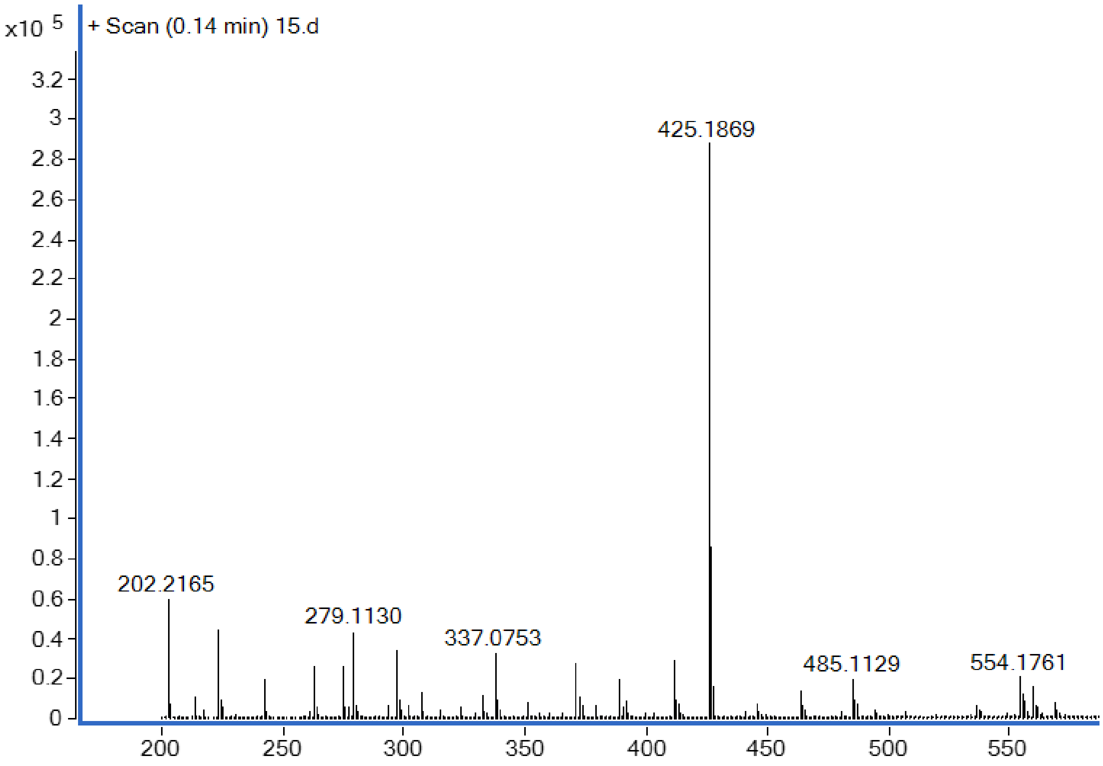

Figure S12 HRMS spectrum of compound **12d**

^{1}H NMR of compound 12e

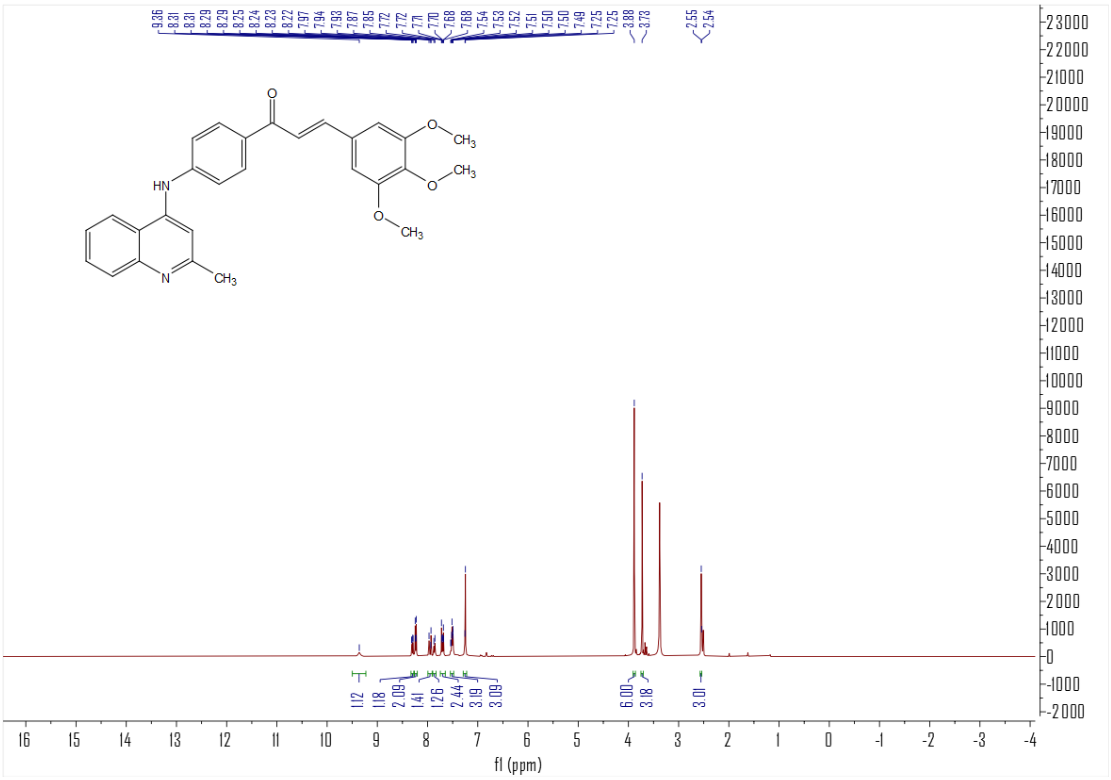

Figure S13 ^{1}H NMR spectrum of compound **12e**

^{13}C NMR of compound 12e

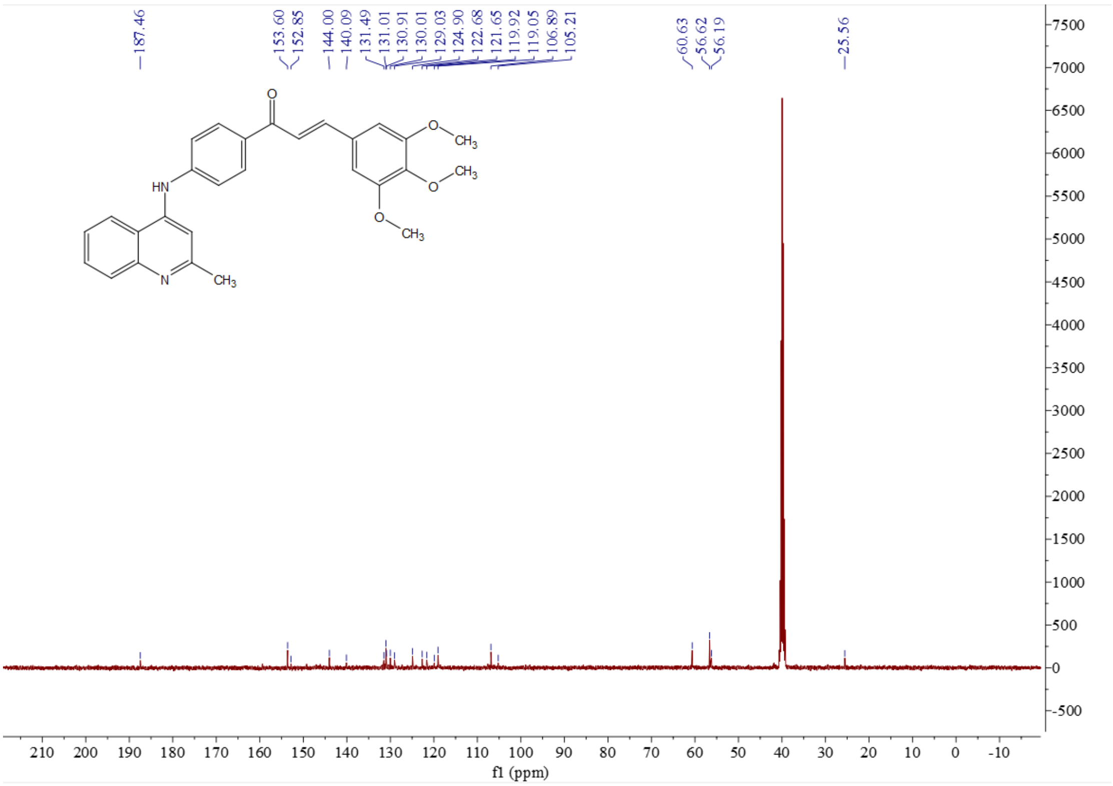

Figure S14 ^{13}C NMR spectrum of compound **12e**

HNMRS spectrum of compound 12e

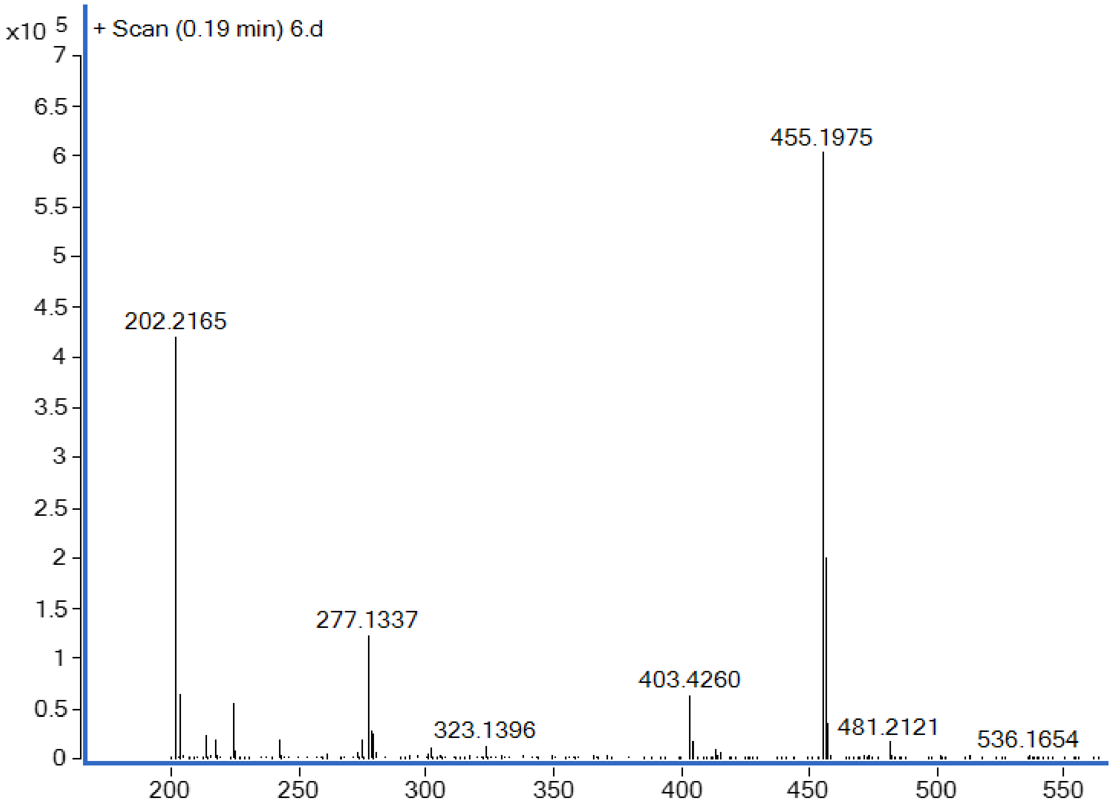

Figure S15 HRMS spectrum of compound **12e**

^{1}H NMR of compound 12f

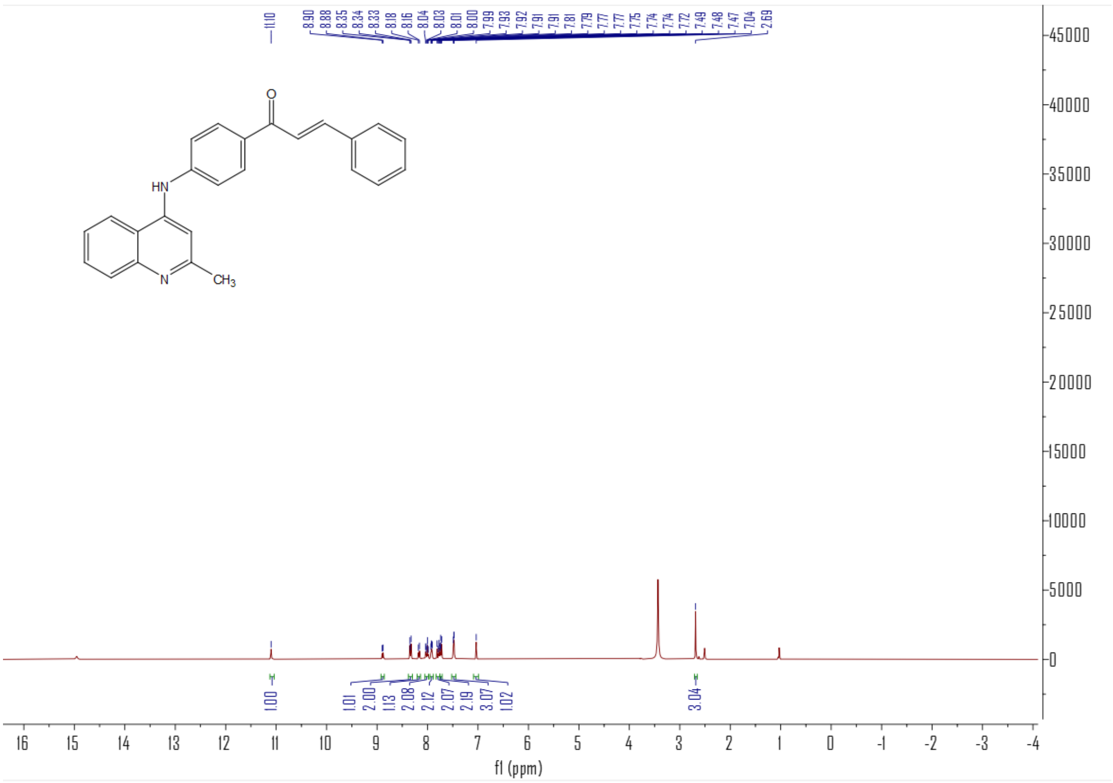

Figure S16 ^{1}H NMR spectrum of compound **12f**

^{13}CNMR of compound 12f

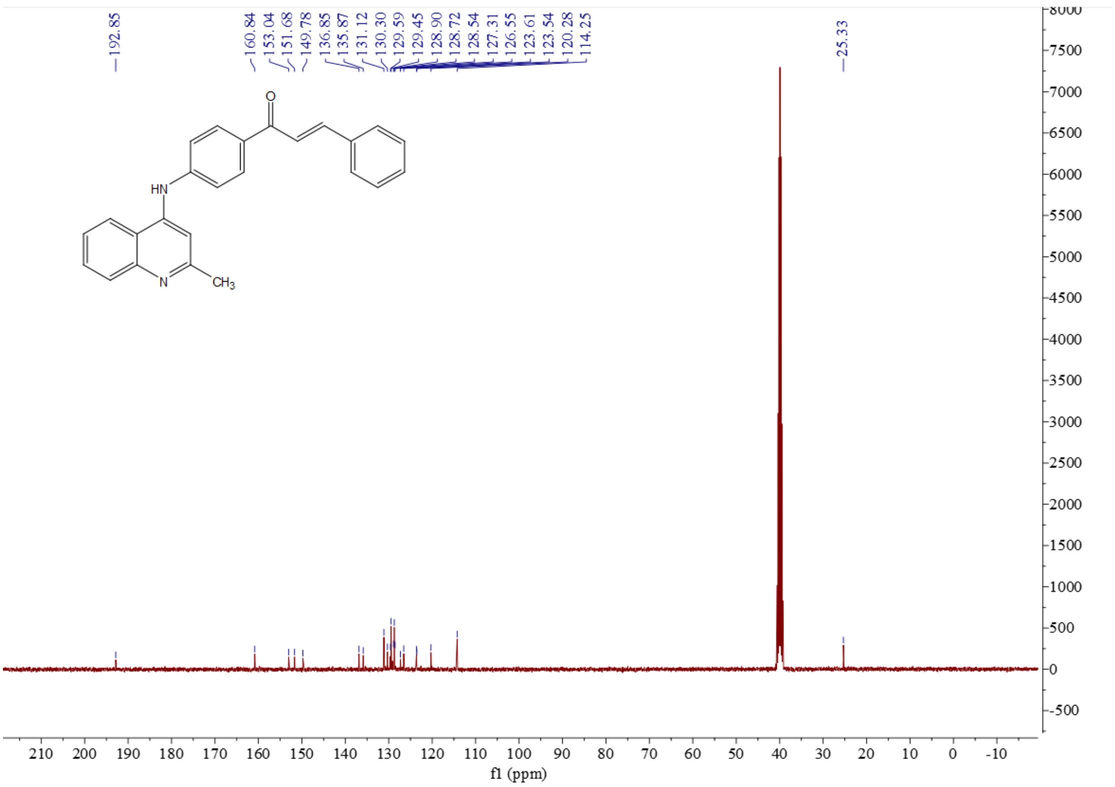

Figure S17 ^{13}C NMR spectrum of compound **12f**

HRMS spectrum of compound 12f

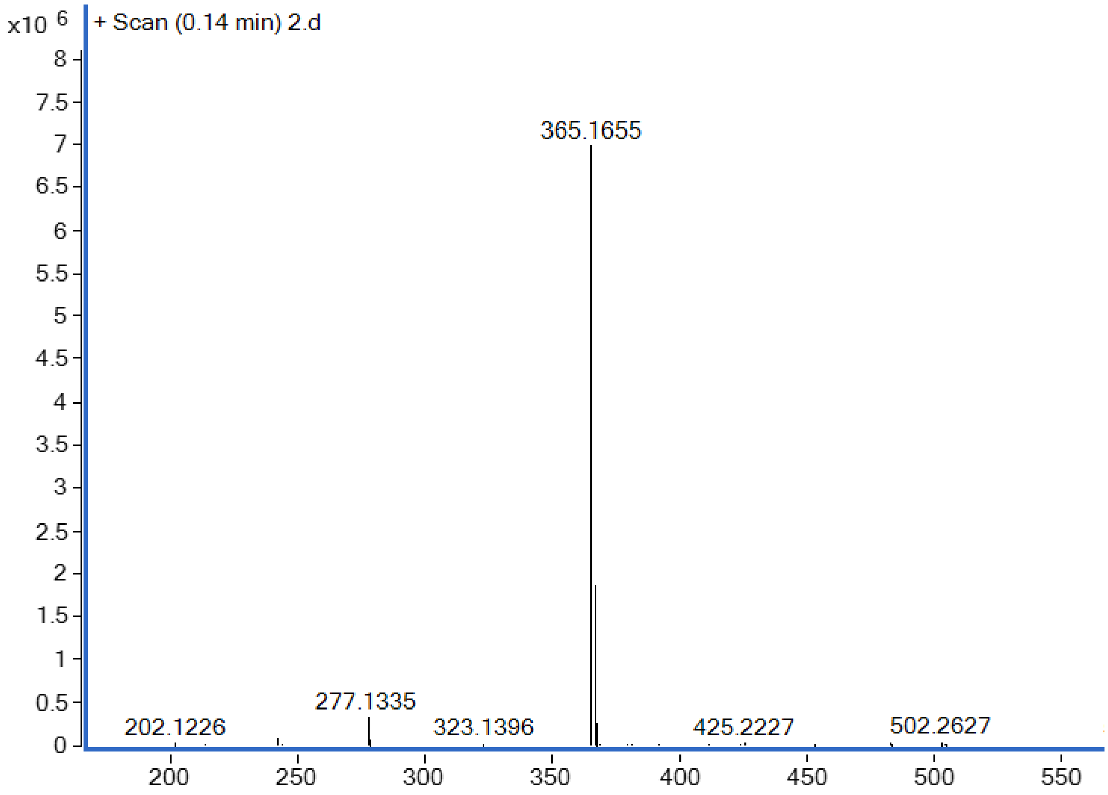

Figure S18 HRMS spectrum of compound **12f**

^{1}H NMR of compound 12g

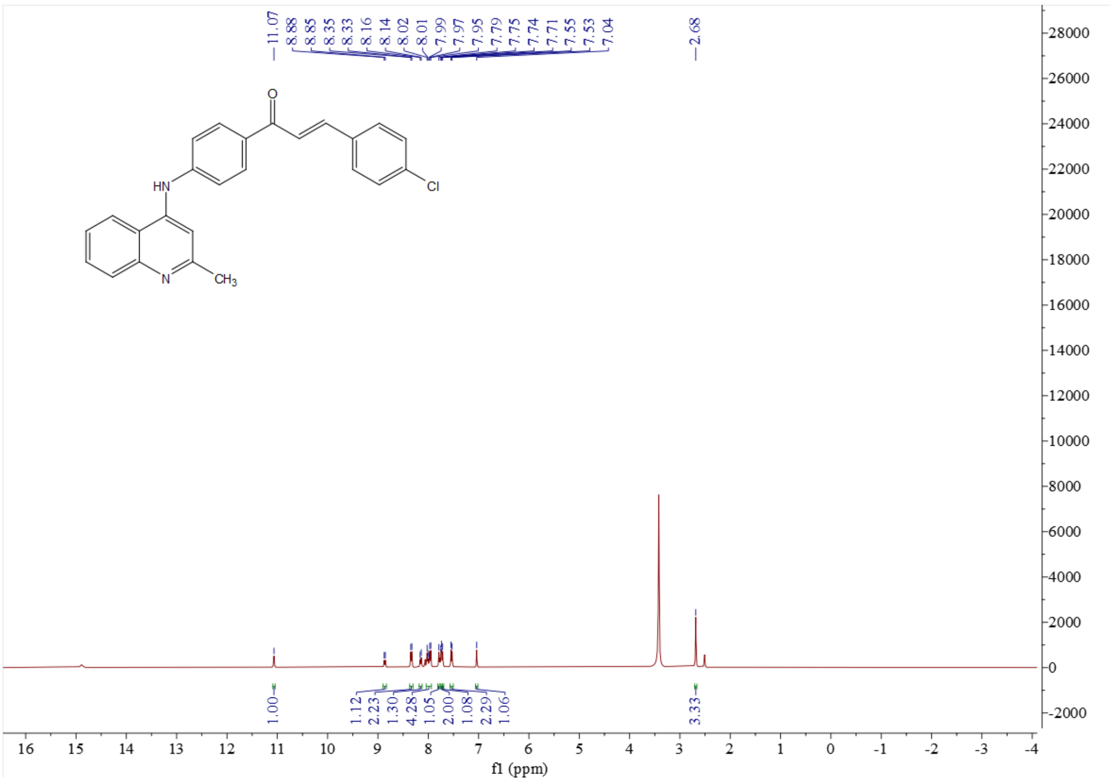

Figure S19 ^{1}H NMR spectrum of compound **12g**

^{13}C NMR of compound 12g

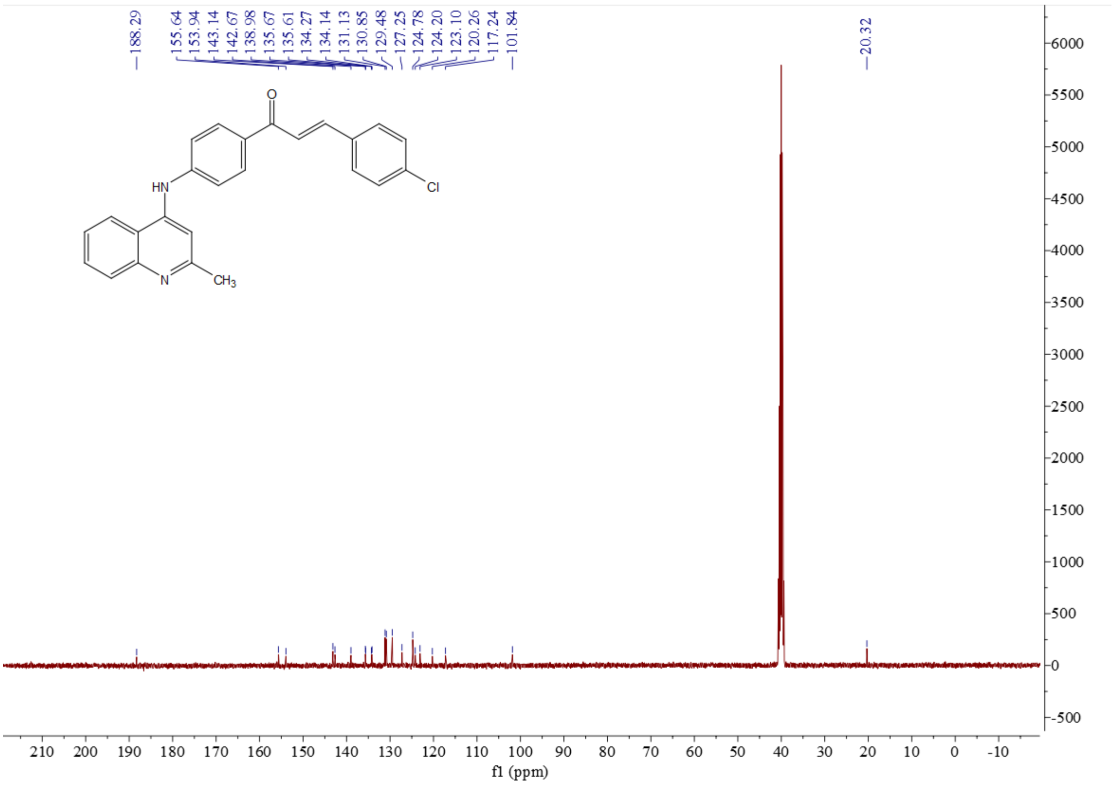

Figure S20 ^{13}C NMR spectrum of compound **12g**

HRMS spectrum of compound 12g

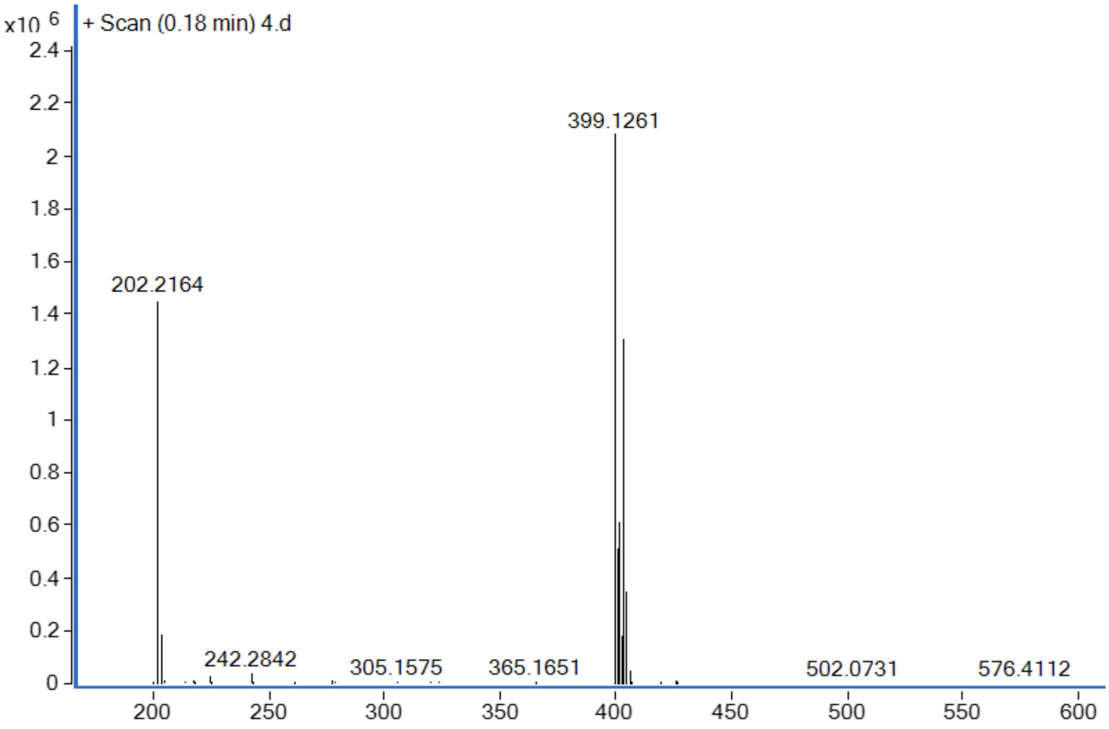

Figure S21 HRMS spectrum of compound **12g**

^{1}H NMR of compound 12h

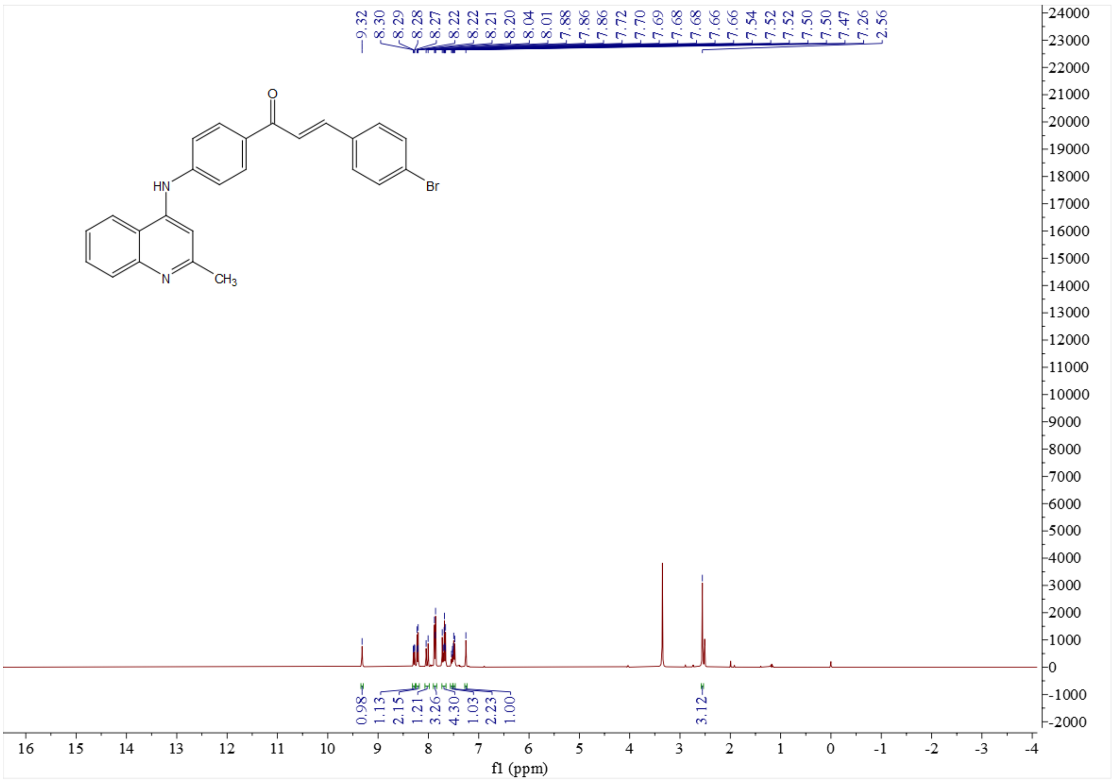

Figure S22 ^{1}H NMR spectrum of compound **12h**

^{13}C NMR of compound 12h

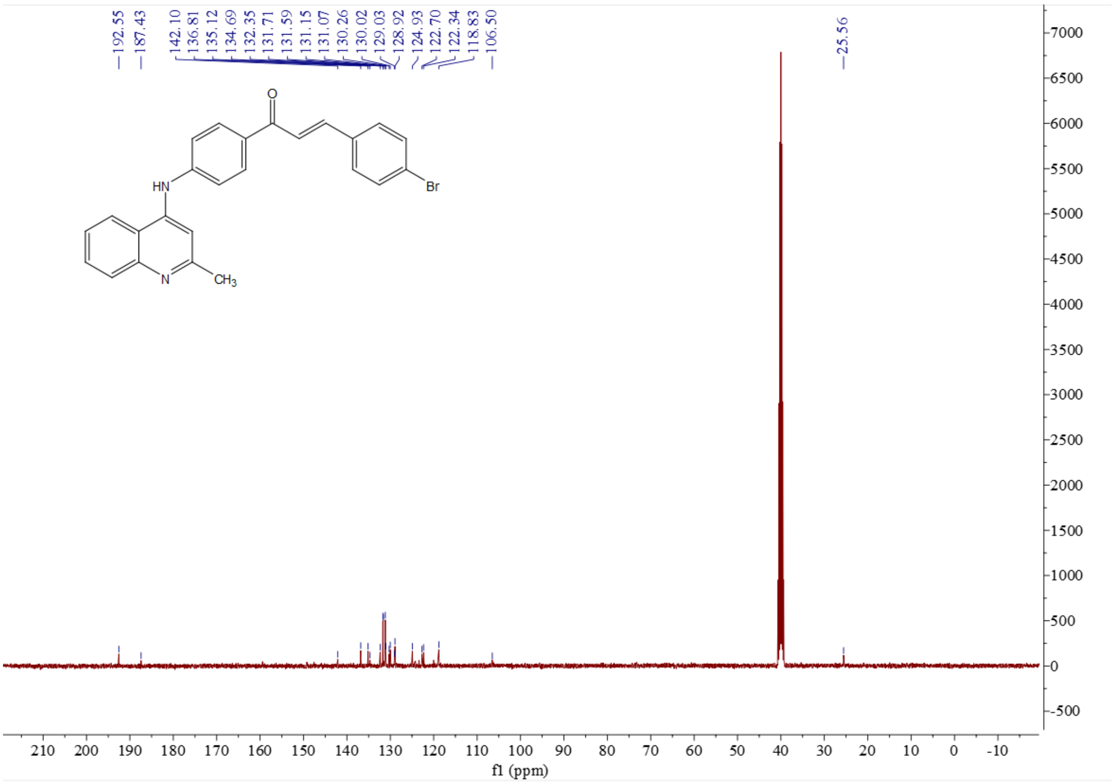

Figure S23 ^{13}C NMR spectrum of compound **12h**

HRMS spectrum of compound 12h

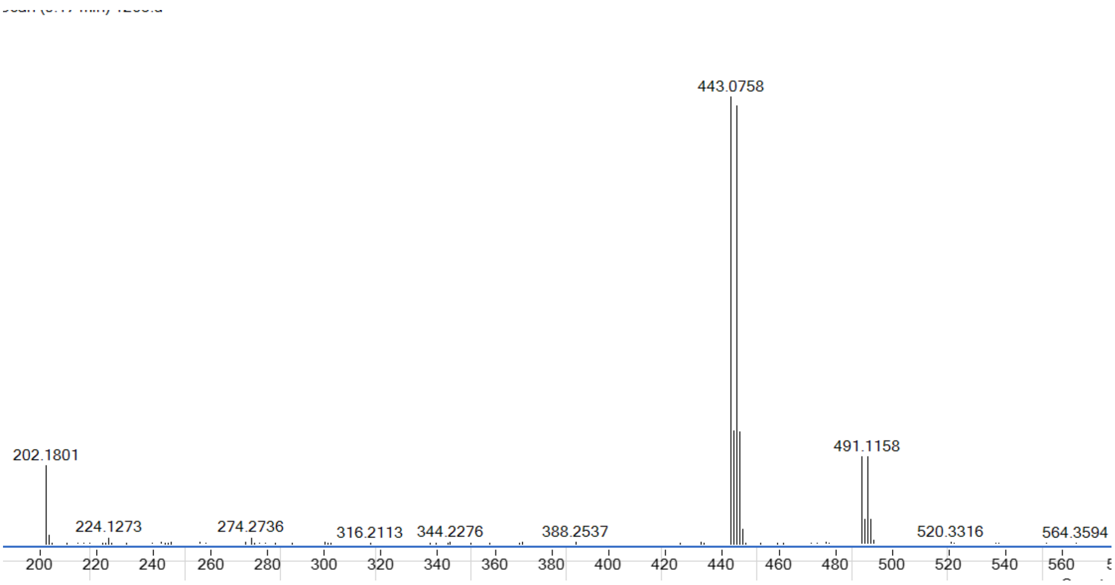

Figure S24 HRMS spectrum of compound **12h**

^{1}H NMR of compound 12i

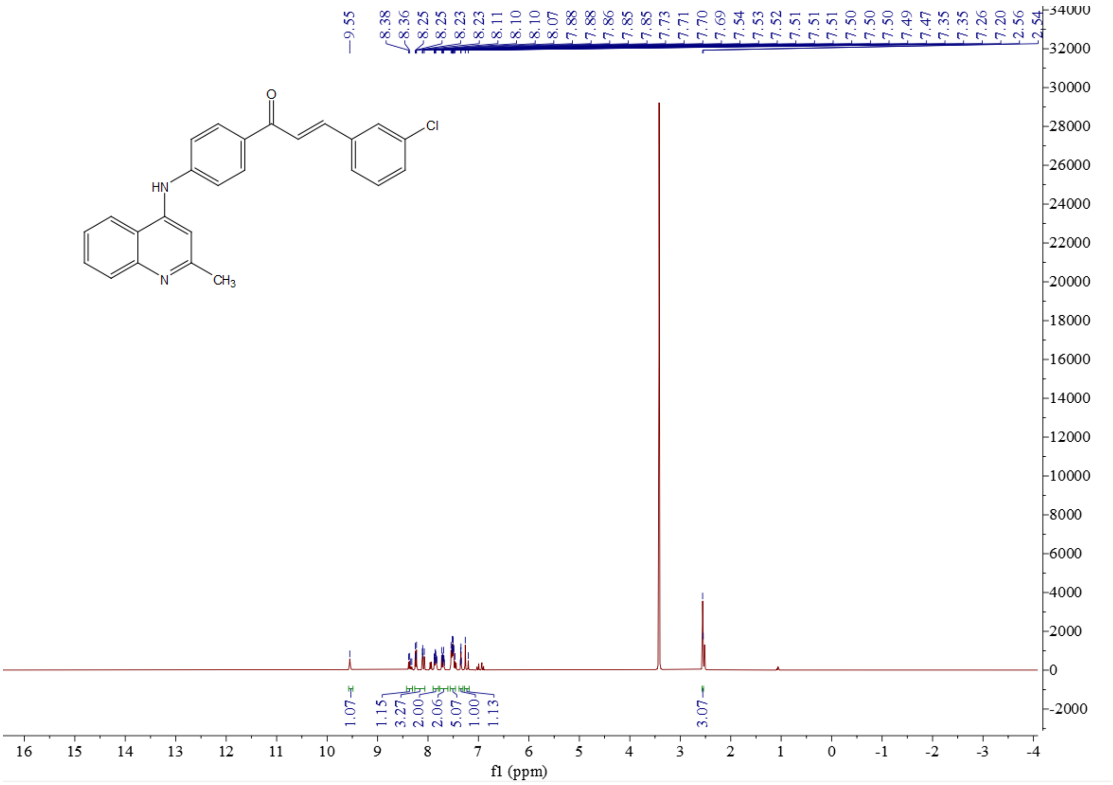

Figure S25 ^{1}H NMR spectrum of compound **12i**

^{13}C NMR of compound 12i

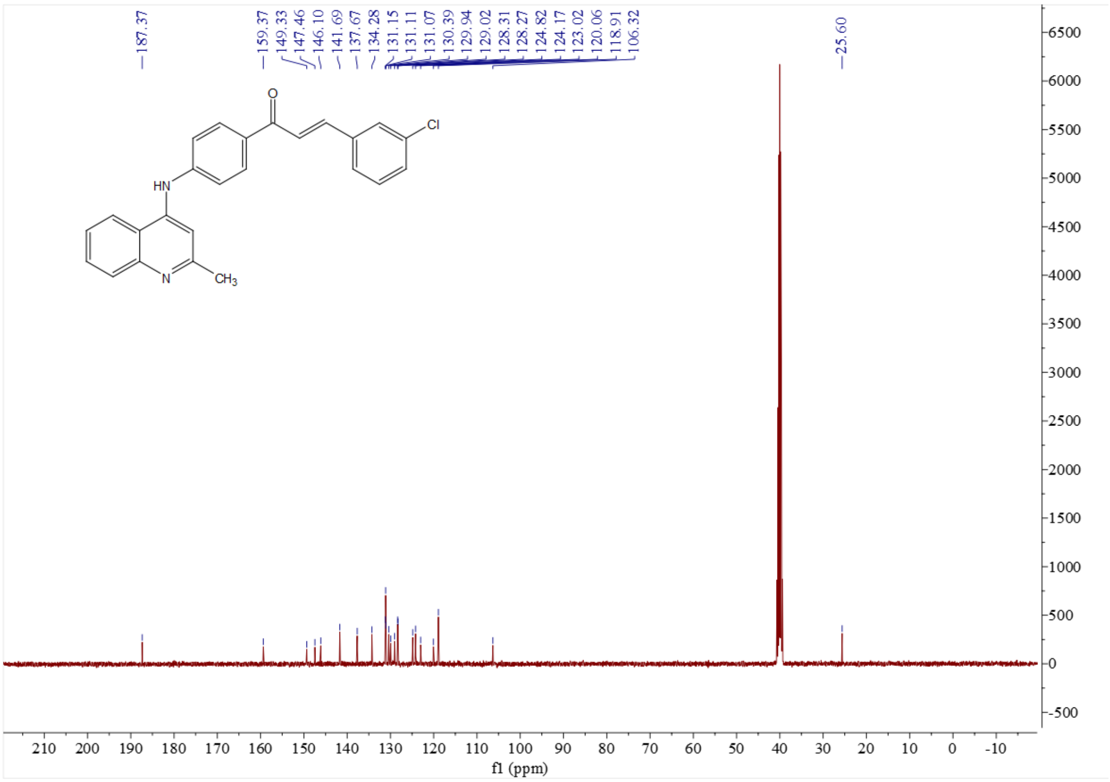

Figure S26 ^{13}C NMR spectrum of compound **12i**

HRMS spectrum of compound 12i

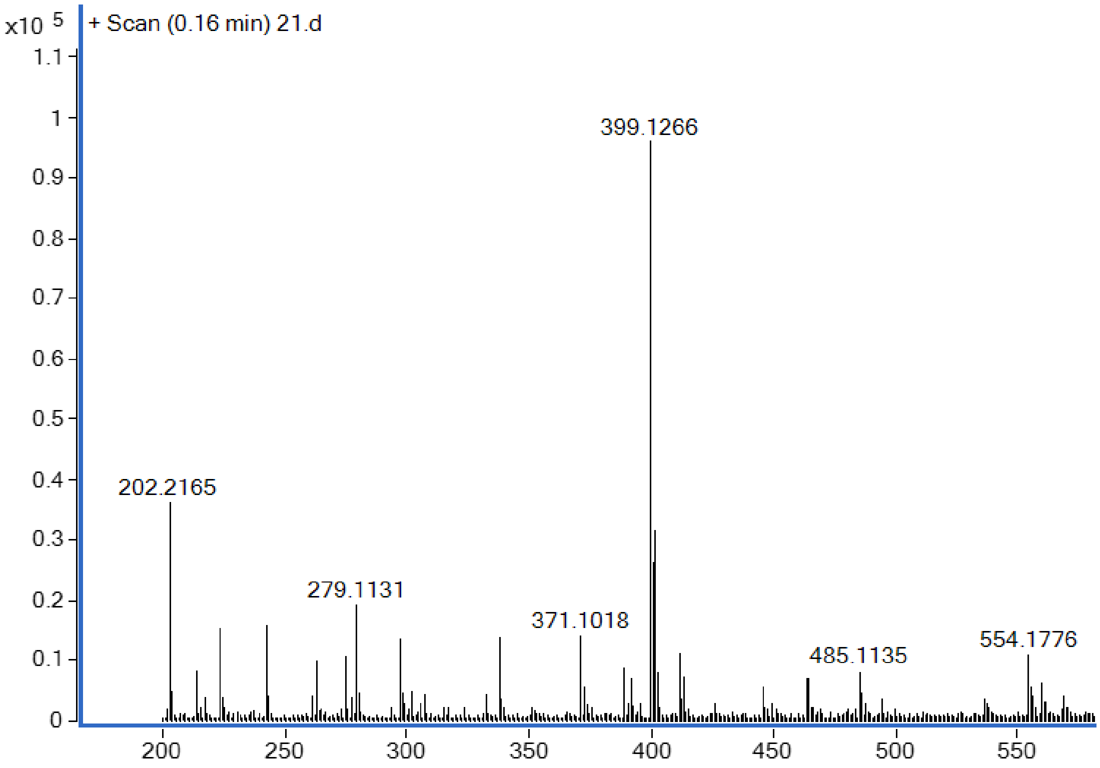

Figure S27 HRMS spectrum of compound **12i**

^{1}H NMR of compound 12j

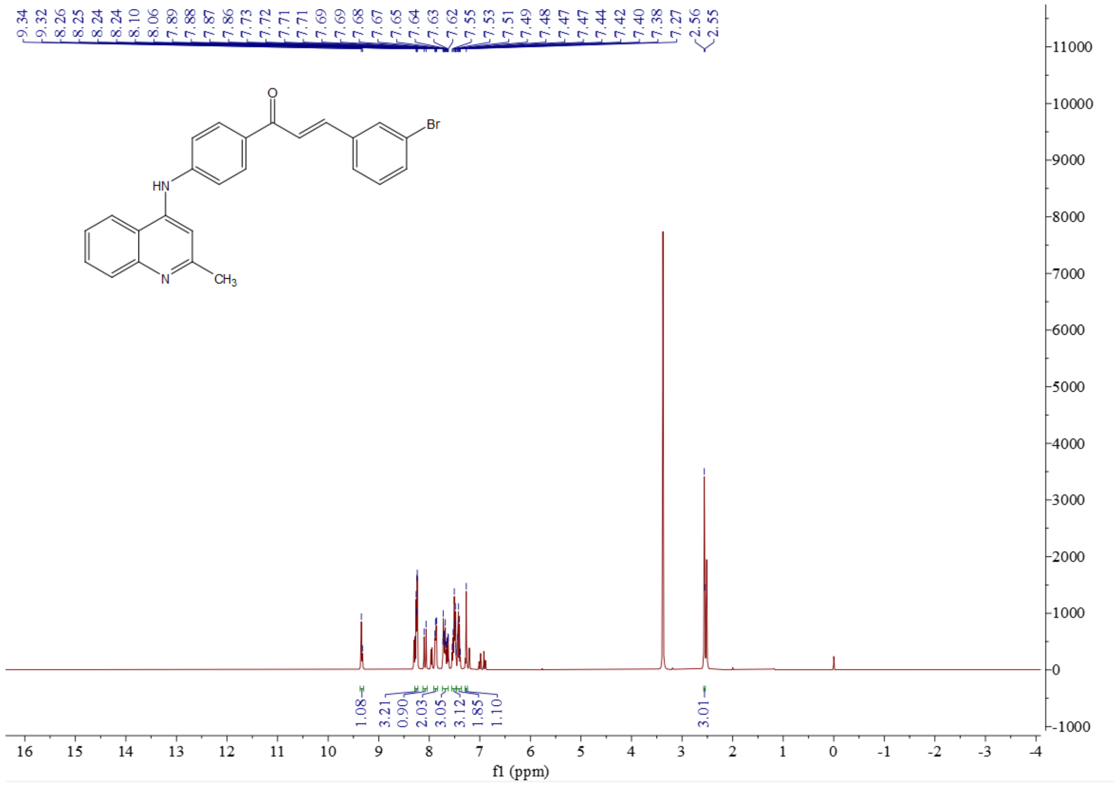

Figure S28 ^{1}H NMR spectrum of compound **12j**

^{13}C NMR of compound 12j

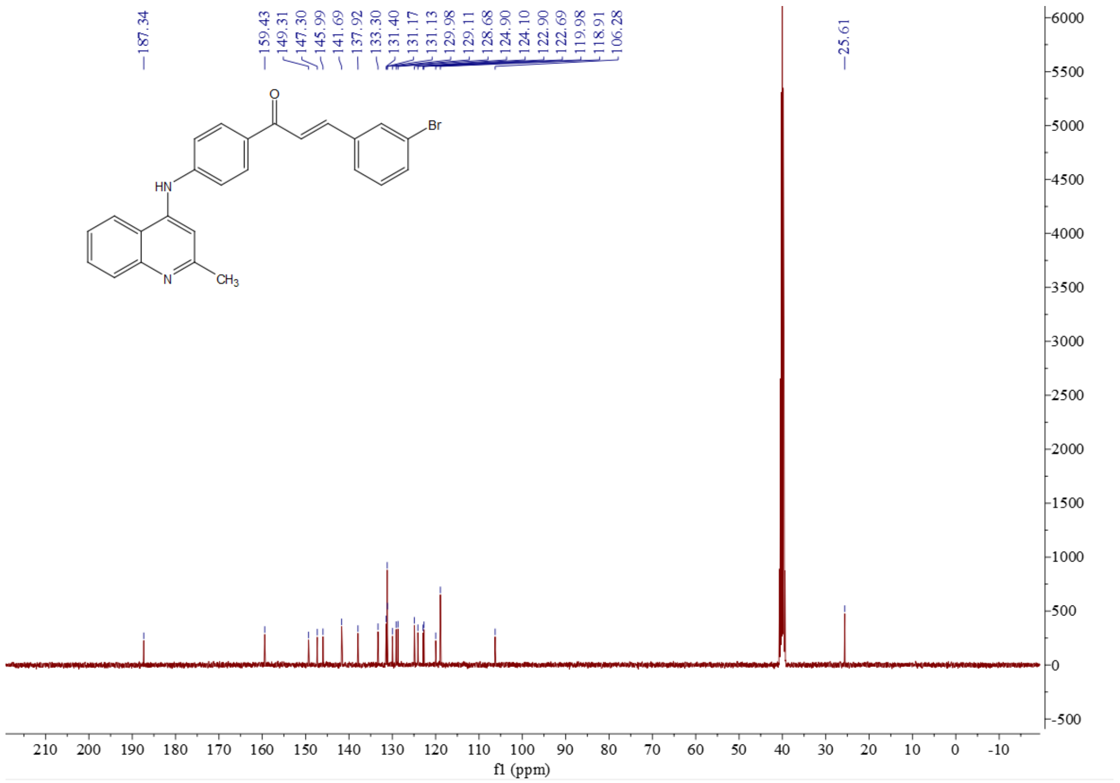

Figure S29 ^{13}C NMR spectrum of compound **12j**

HRMS spectrum of compound 12j

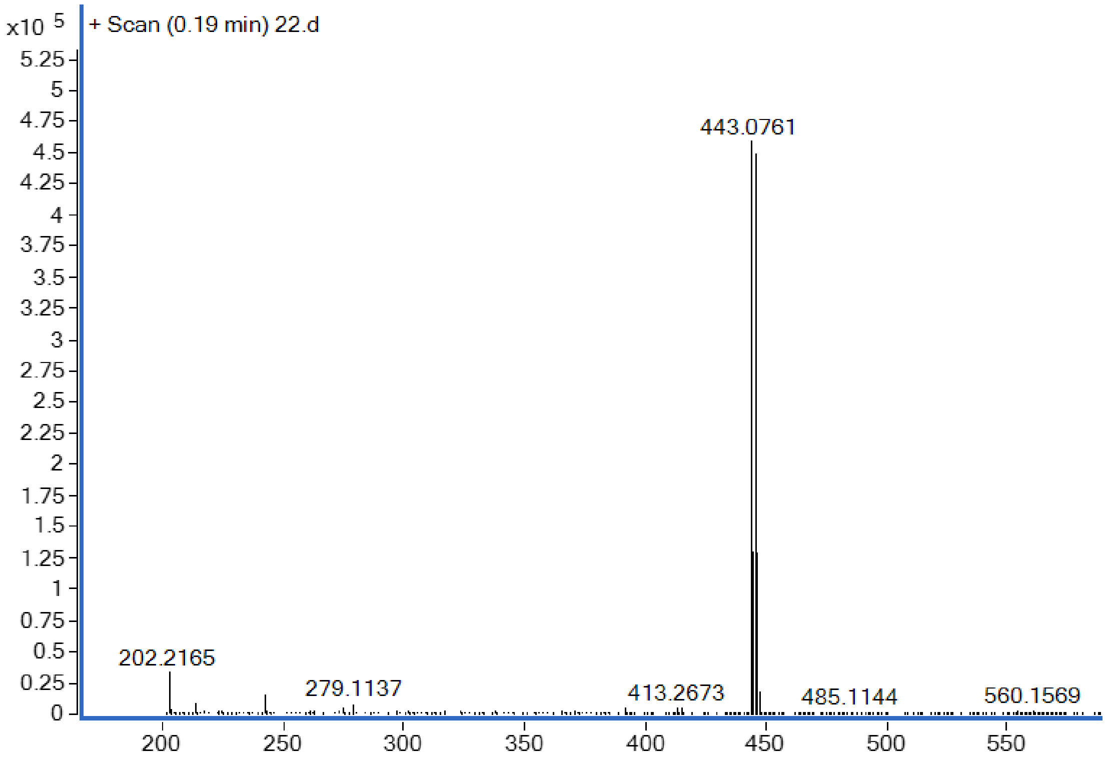

Figure S30 HRMS spectrum of compound **12j**

^{1}H NMR of compound 13a

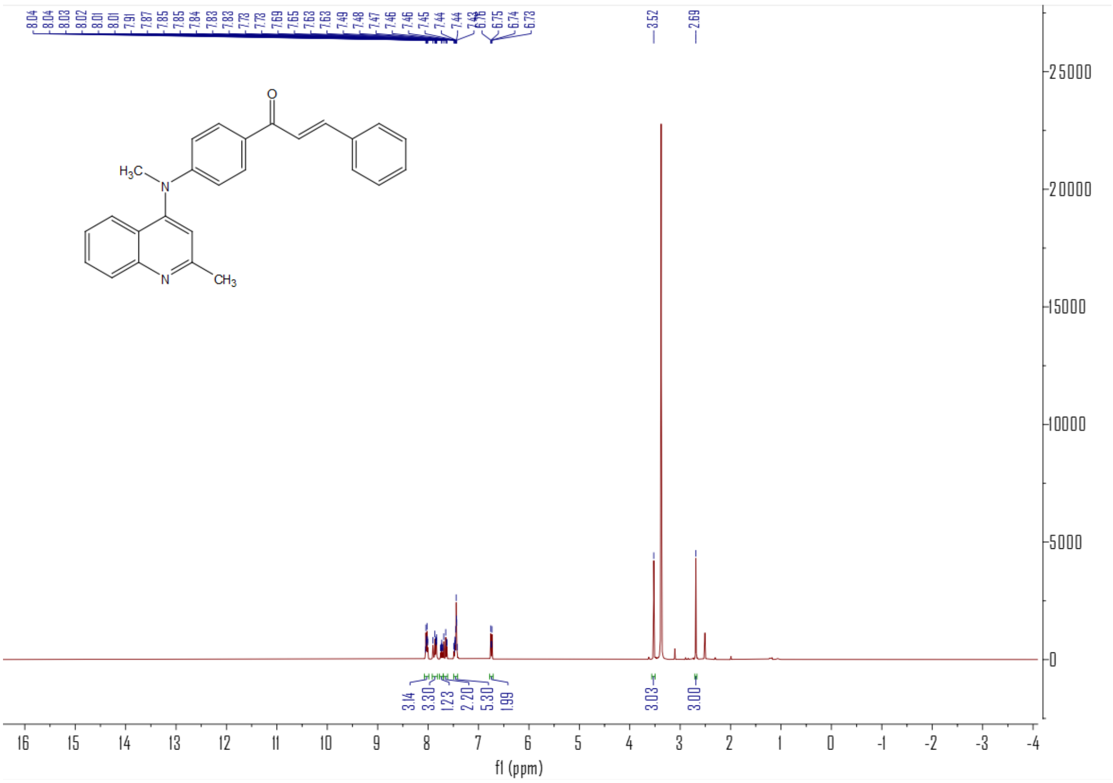

Figure S31 ^{1}H NMR spectrum of compound **13a**

^{13}C NMR of compound 13a

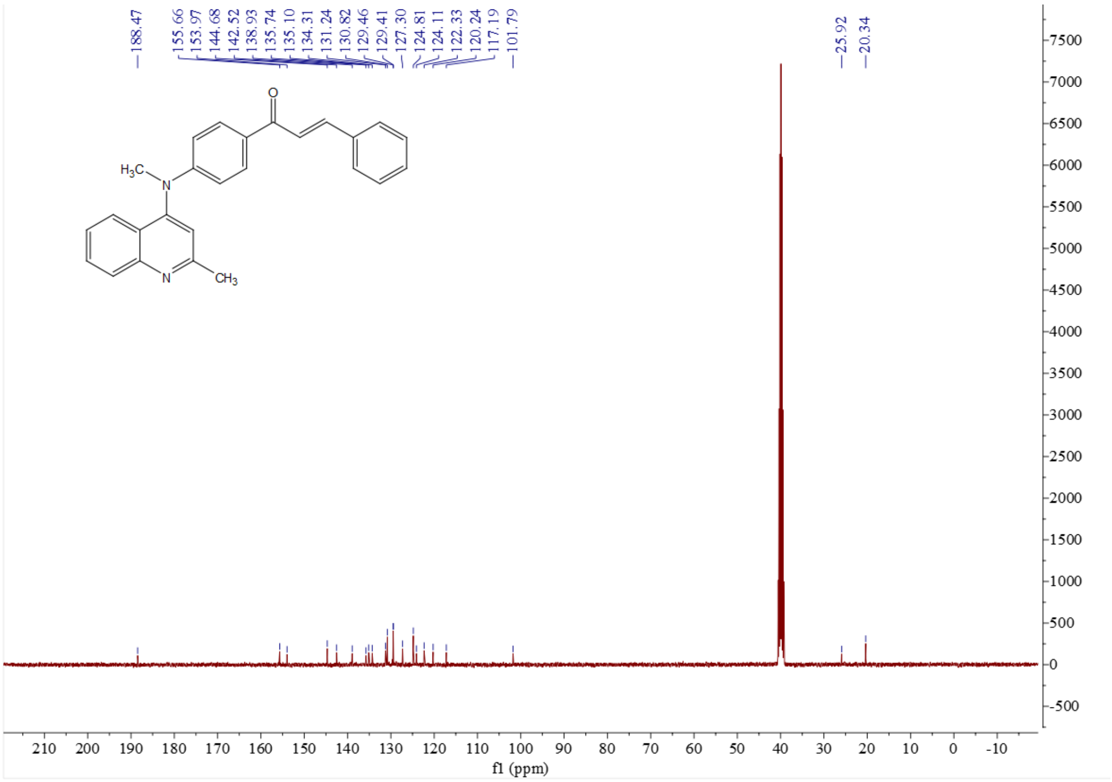

Figure S32 ^{13}C NMR spectrum of compound **13a**

HRMS spectrum of compound 13a

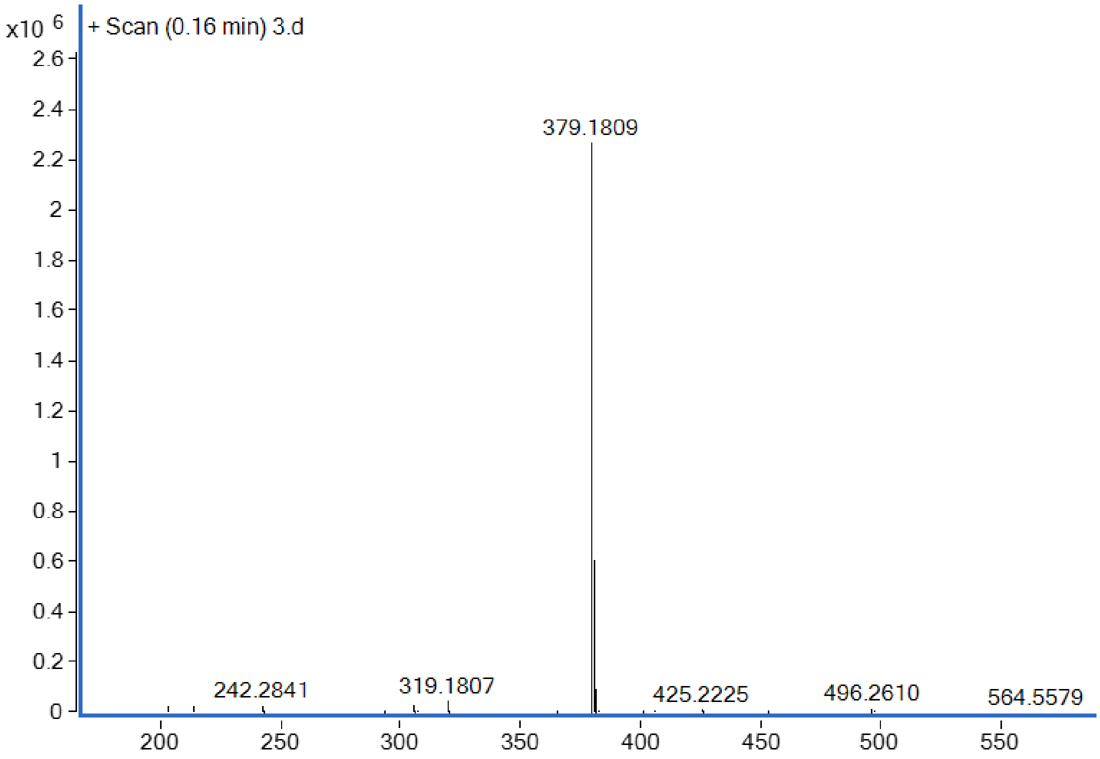

Figure S33 HRMS spectrum of compound **13a**

^{1}H NMR of compound 13b

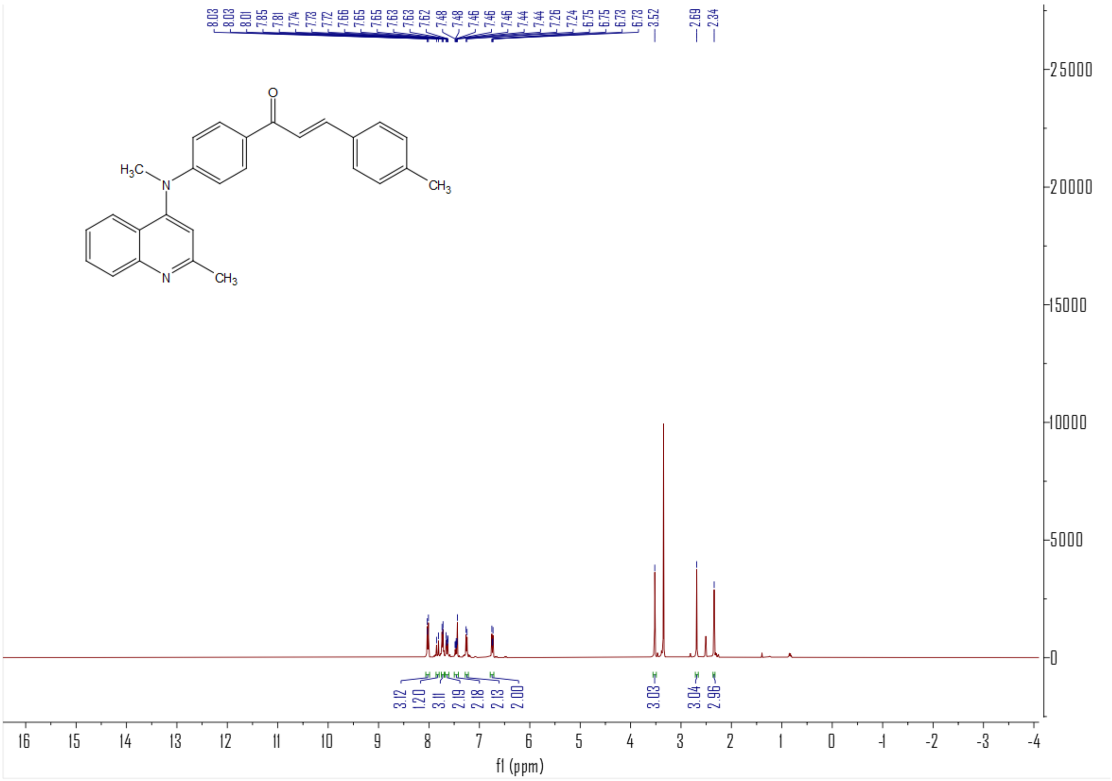

Figure S34 ^{1}H NMR spectrum of compound **13b**

^{13}C NMR of compound 13b

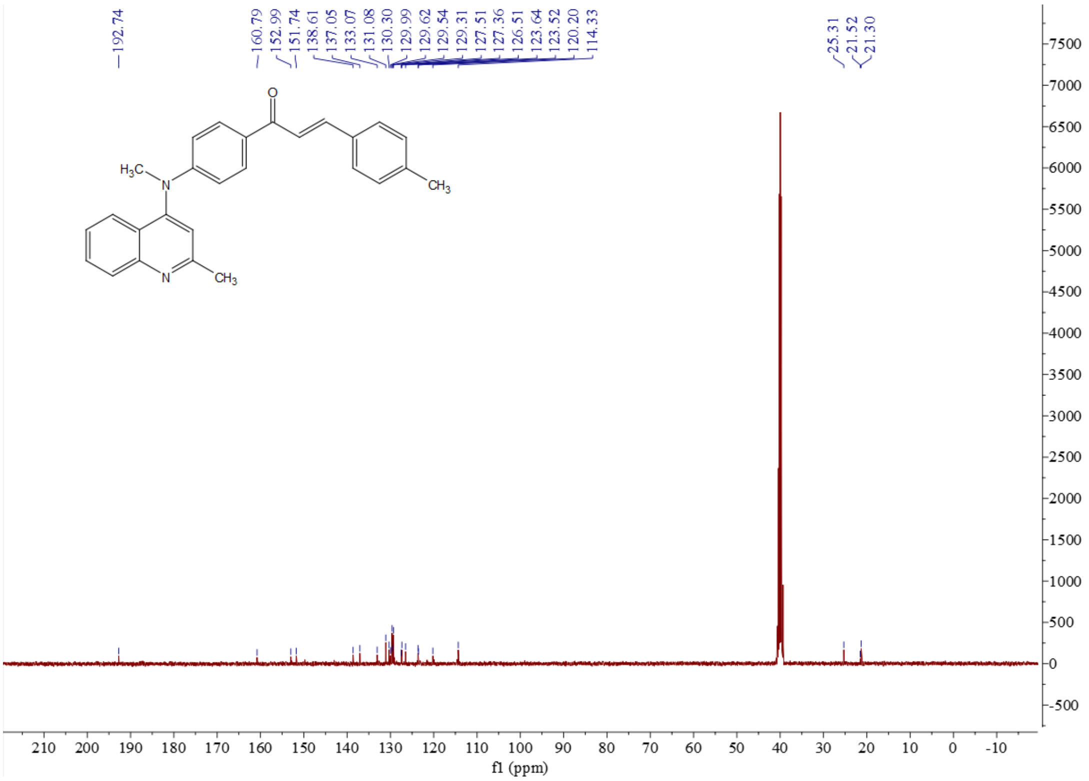

Figure S35 ^{13}C NMR spectrum of compound **13b**

HRMS spectrum of compound 13b

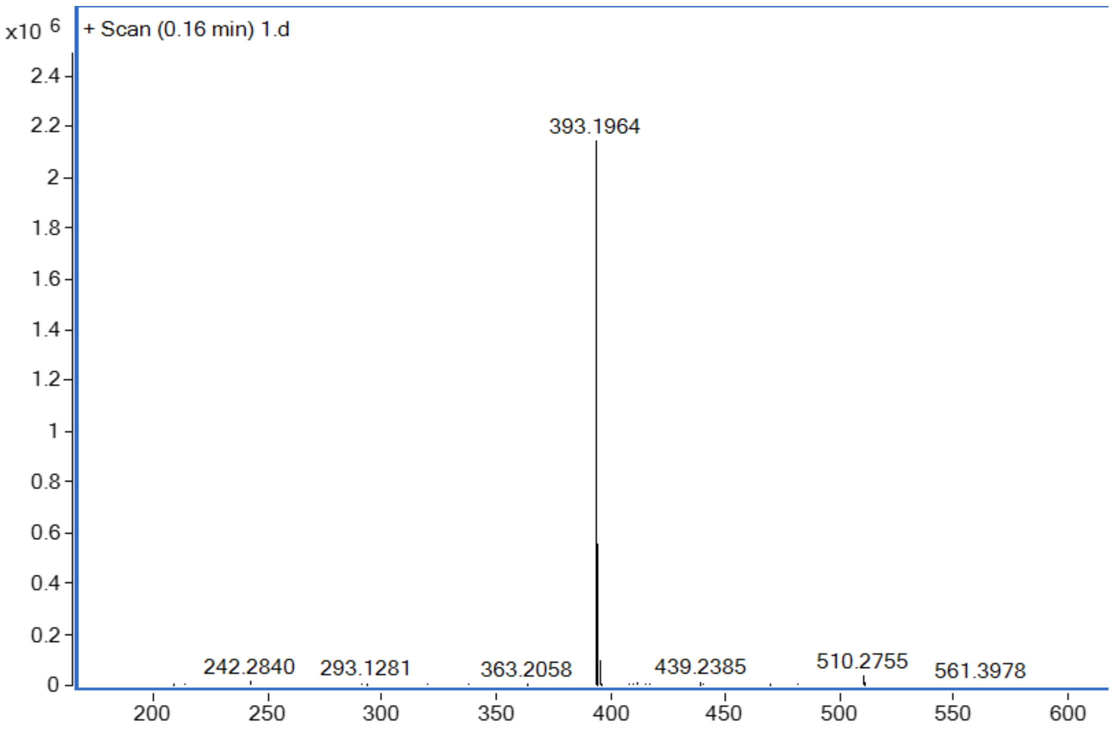

Figure S36 HRMS spectrum of compound **13b**

^{1}H NMR of compound 13c

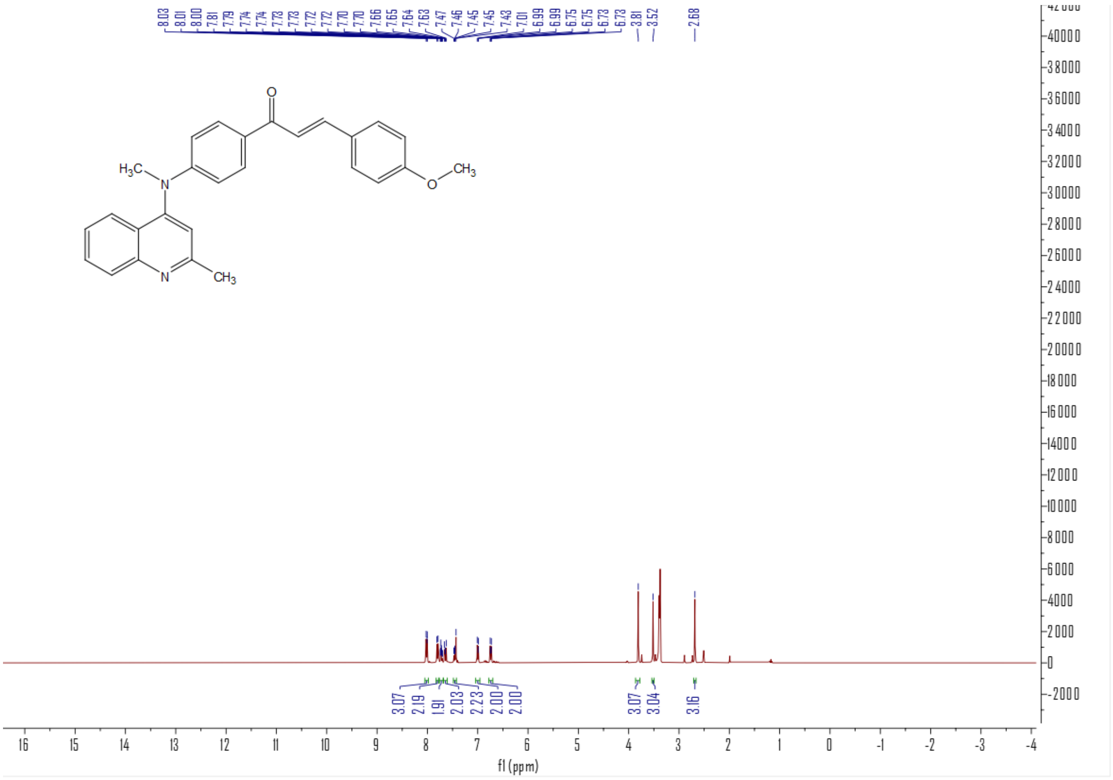

Figure S37 ^{1}H NMR spectrum of compound **13c**

^{13}C NMR of compound 13c

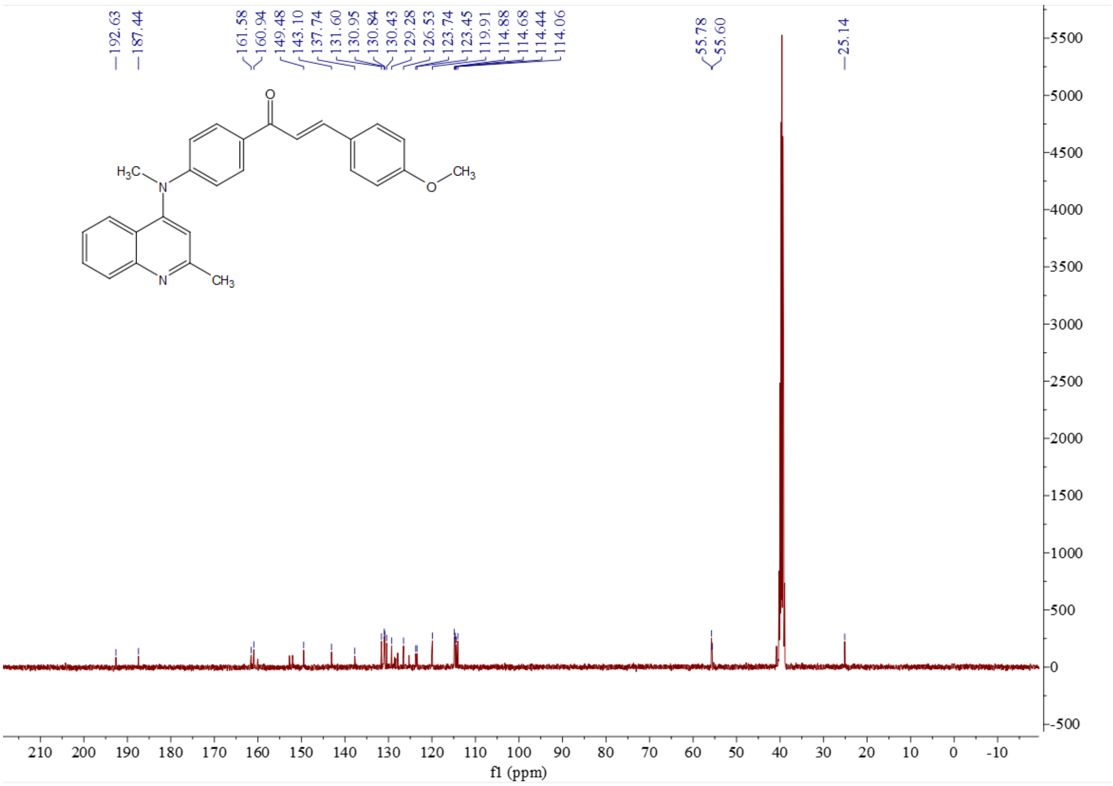

Figure S38 ^{13}C NMR spectrum of compound **13c**

HRMS spectrum of compound 13c

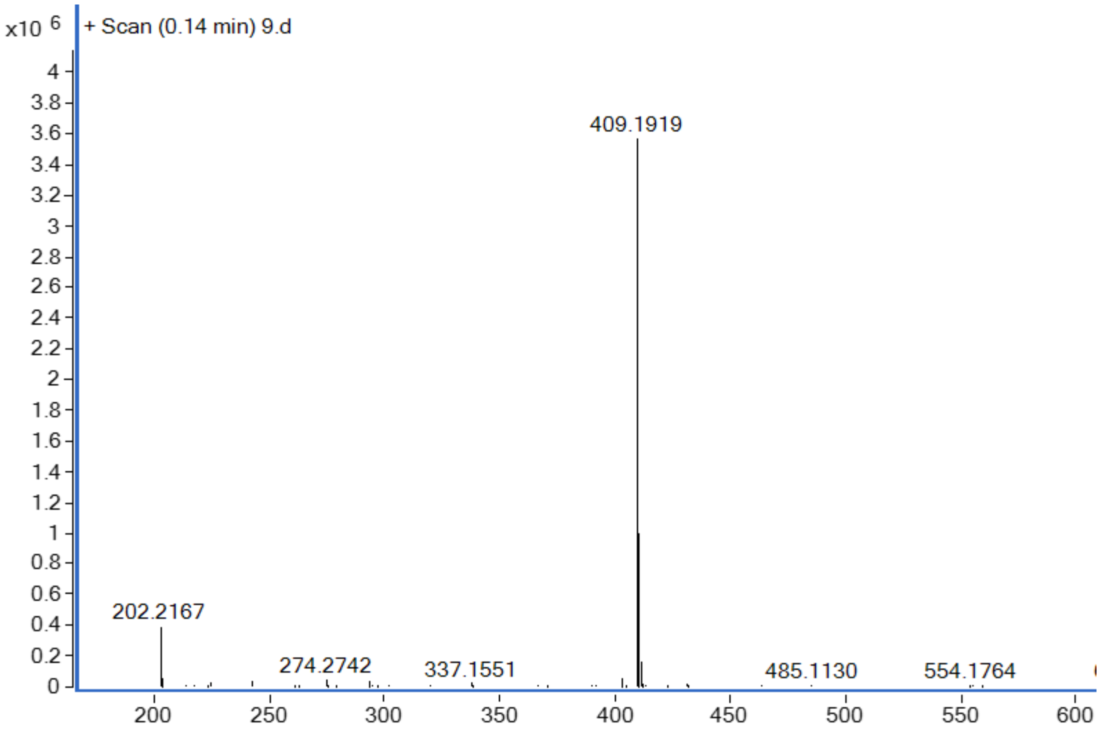

Figure S39 HRMS spectrum of compound **13c**

^{1}H NMR of compound 13d

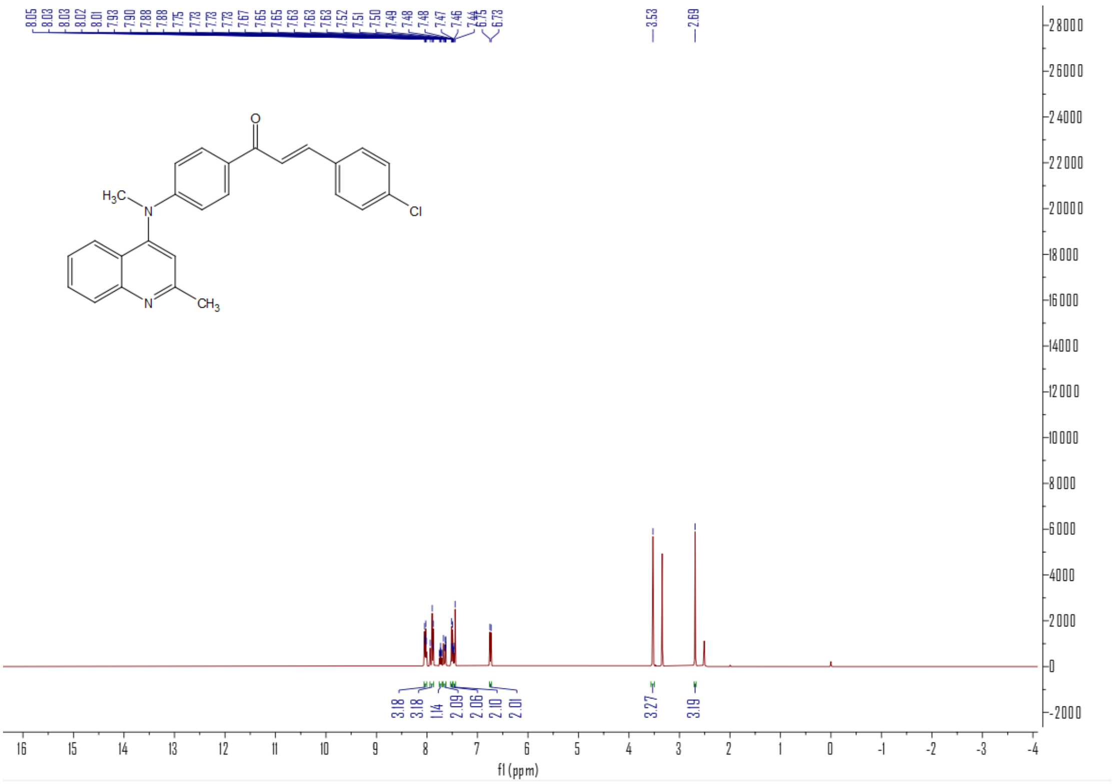

Figure S40 ^{1}H NMR spectrum of compound **13d**

^{13}C NMR of compound 13d

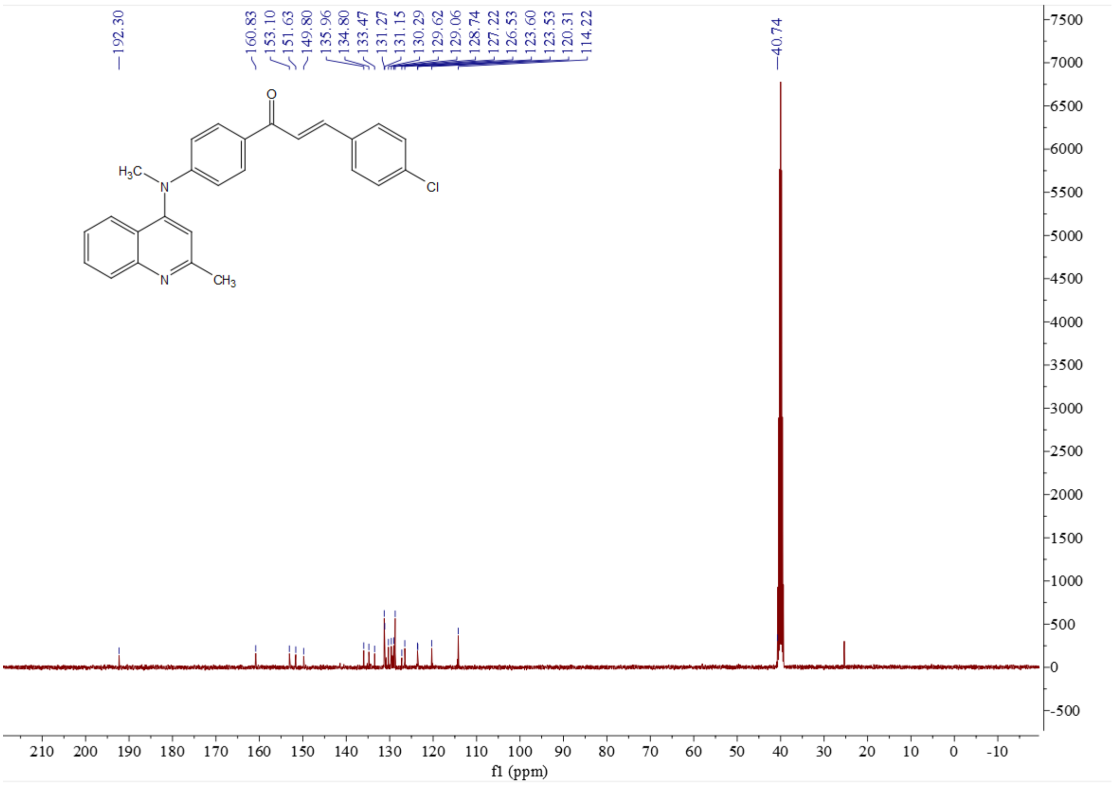

Figure S41 ^{13}C NMR spectrum of compound **13d**

HRMS spectrum of compound 13d

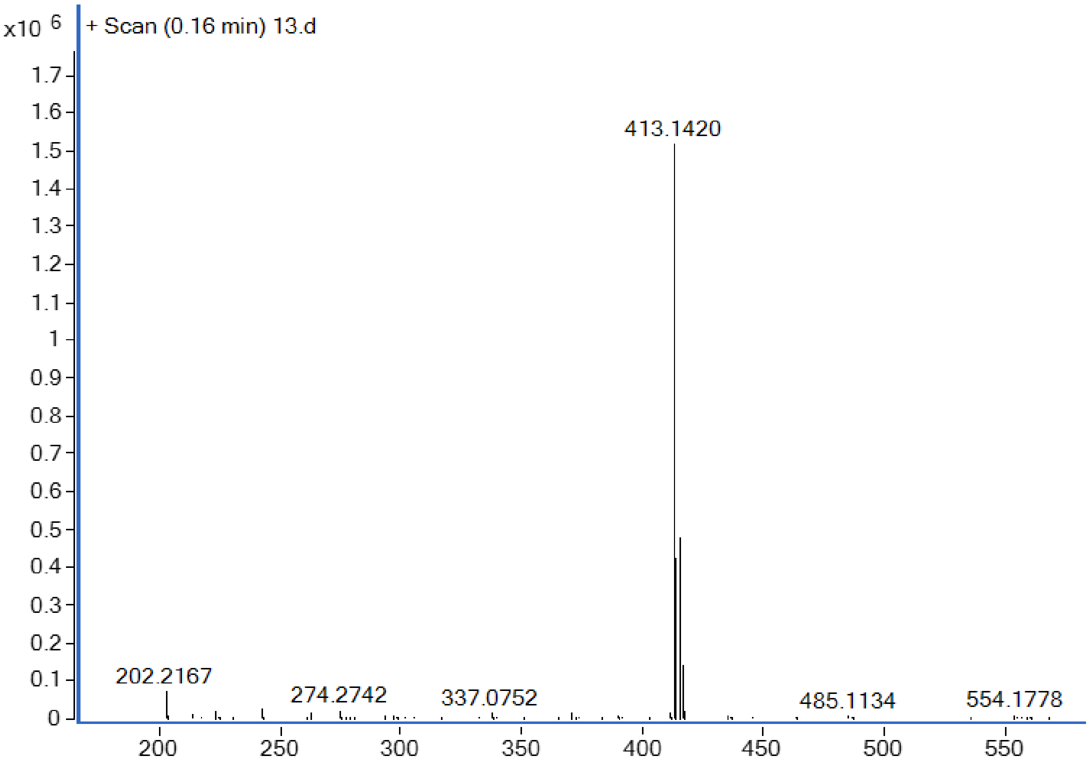

Figure S42 HRMS spectrum of compound **13d**

^{1}H NMR of compound 13e

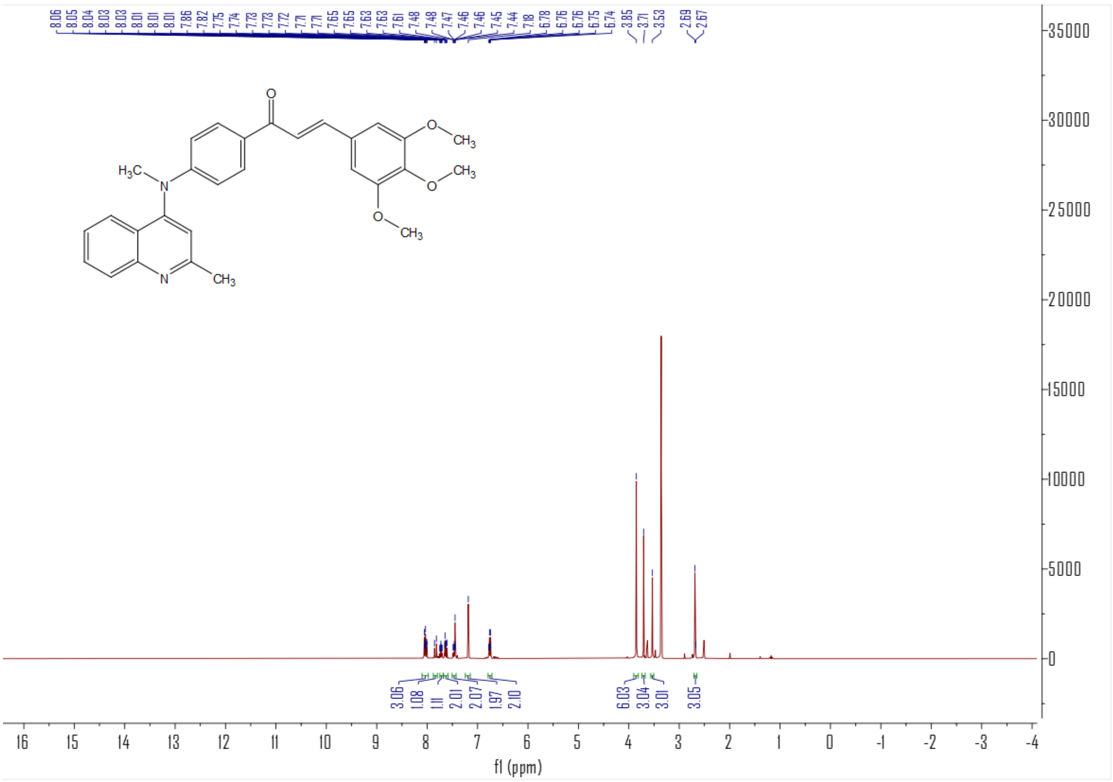

Figure S43 ^{1}H NMR spectrum of compound **13e**

^{13}C NMR of compound 13e

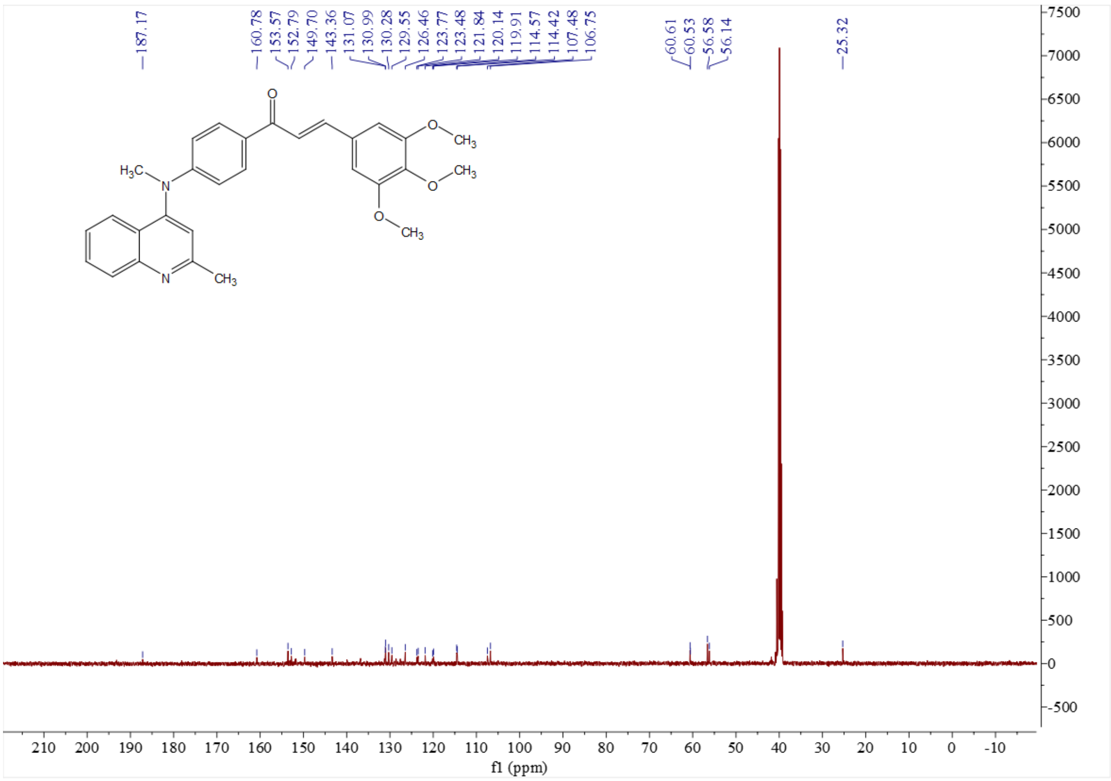

Figure S44 ^{13}C NMR spectrum of compound **13e**

HRMS spectrum of compound 13e

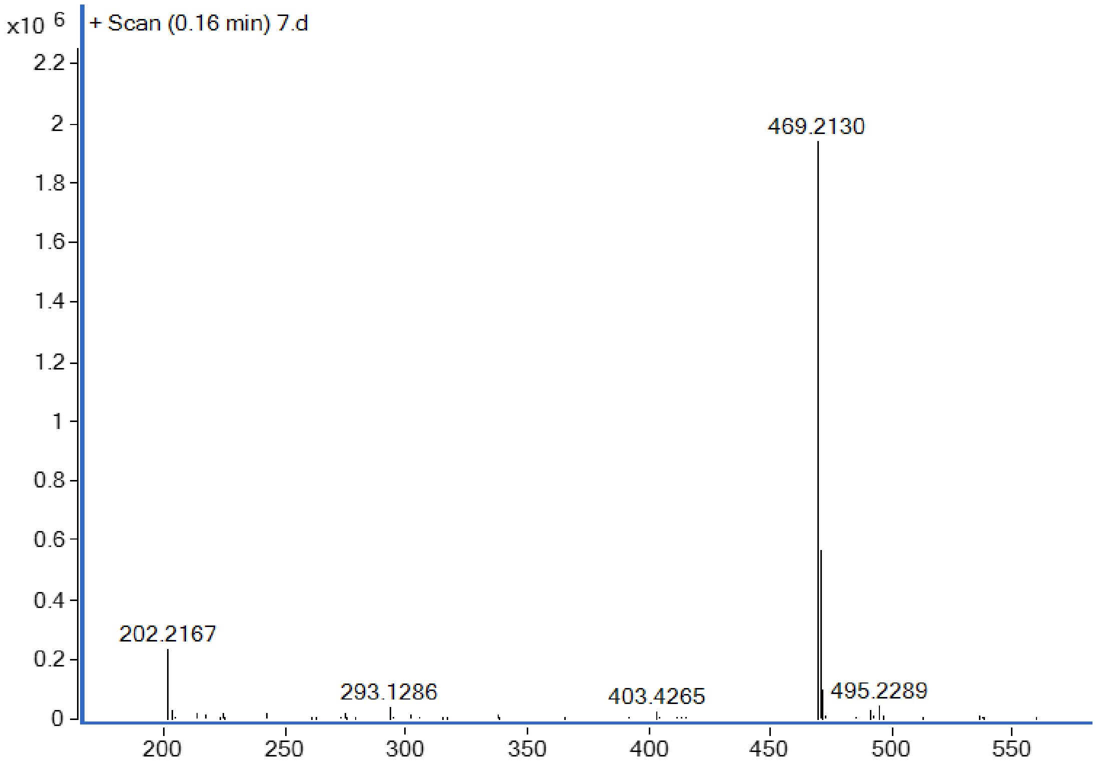

Figure S45 HRMS spectrum of compound **13e**

^{1}H NMR of compound 13f

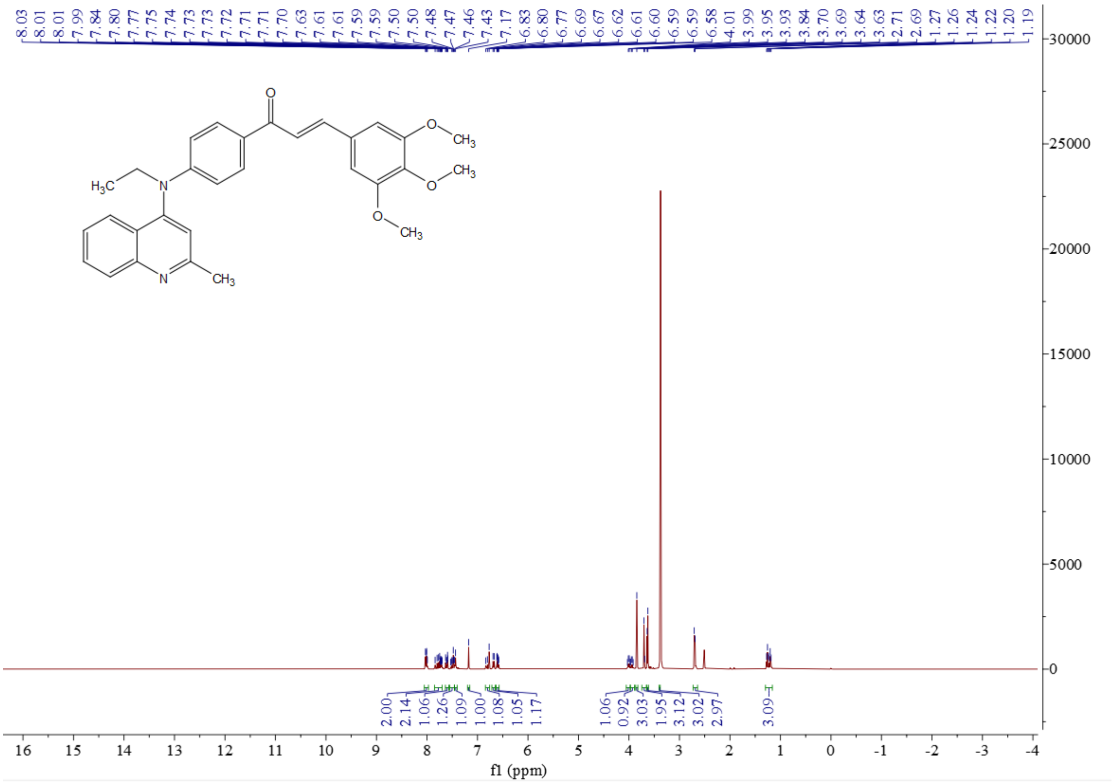

Figure S46 ^{1}H NMR spectrum of compound **13f**

^{13}C NMR of compound 13f

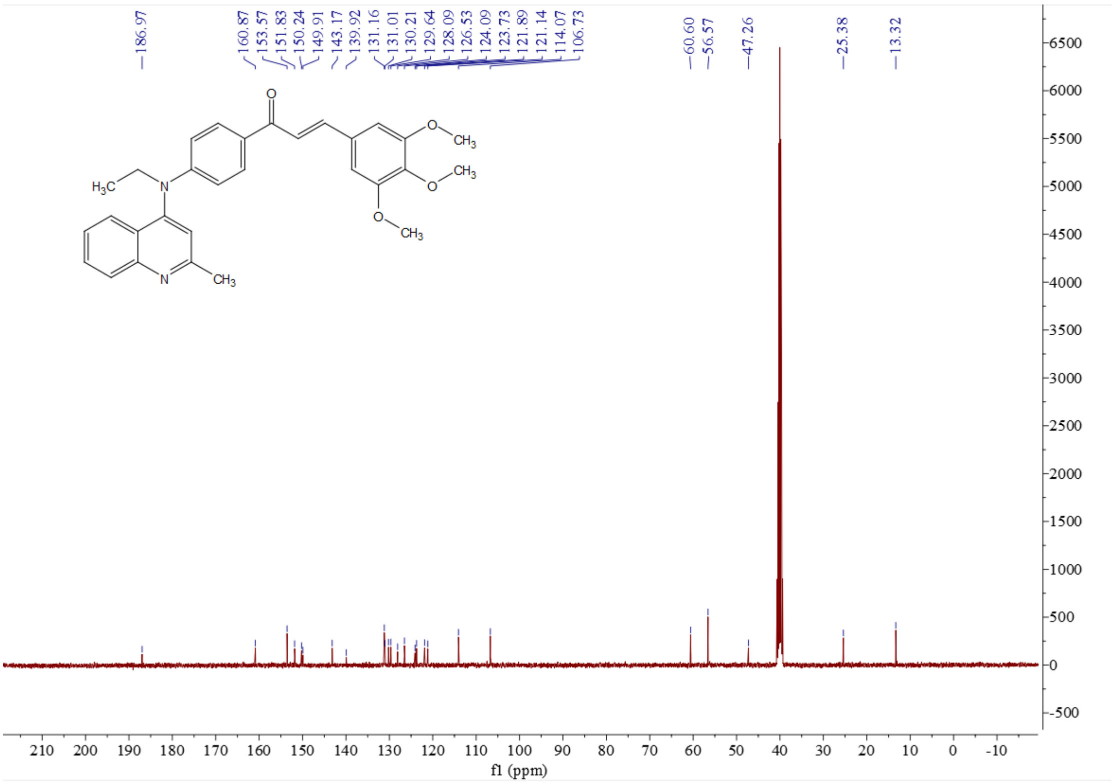

Figure S47 ^{13}C NMR spectrum of compound **13f**

HRMS spectrum of compound 13f

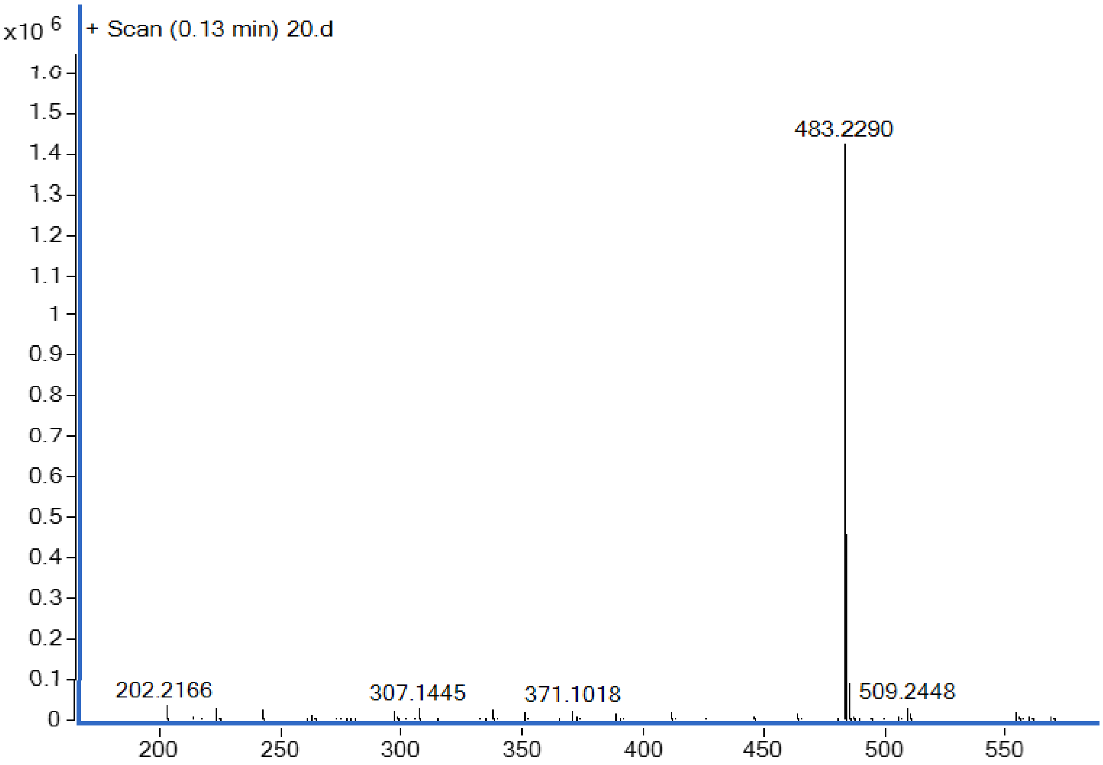

Figure S48 HRMS spectrum of compound **13f**
